# Bidirectional Phase Separation Spinning Under Natural Drying to Prepare Aerogel Fibers for Thermal Insulation

**DOI:** 10.1002/advs.202505306

**Published:** 2025-07-18

**Authors:** Jiaxin Shen, Shisheng Hou, Chen Li, Kuibo Yin, Li Zhong, Hengchang Bi, Litao Sun

**Affiliations:** ^1^ SEU‐FEI Nano‐Pico Center, Key Laboratory of MEMS of the Ministry of Education, Collaborative Innovation Center for Micro/Nano Fabrication, Device and System Southeast University Nanjing 210096 P. R. China; ^2^ In Situ Devices Center, School of Integrated Circuits East China Normal University Dongchuan Road Shanghai 200241 P. R. China; ^3^ Chongqing Key Laboratory of Precision Optics Chongqing Institute of East China Normal University Chongqing 401120 P. R. China

**Keywords:** aerogel fiber, bidirectional phase separation, coaxial wet spinning, energy crisis, thermal insulation

## Abstract

Aerogel fibers have emerged as a promising solution in thermal insulation. Yet, the current preparation methods pose challenges: they are either inefficient for continuous production or involve energy‐intensive and time‐consuming steps like freeze‐drying or supercritical drying, thus restricting their practical utility. Here, a bidirectional phase separation spinning technique is introduced for continuous and rapid production (360 m h^−1^) of aerogel fibers at any desired length. Importantly, fibers can be directly dried at room temperature, eliminating the need for energy‐intensive freeze‐drying or other additional steps, significantly reducing energy consumption and processing time. Aerogel fibers produced using this method exhibit remarkable properties: low density (0.18 g cm^−^^3^), high porosity (84%), and broad operational temperature range (−20 to 120 °C). Notably, the thermal insulation mat composed of these aerogel fibers achieves an exceptionally low thermal conductivity (31 ± 1.2 mW/(mK)). This work provides a novel strategy for the preparation of aerogel fibers, achieving energy savings and efficiency, positioning aerogel fibers as a promising alternative for next‐generation textiles.

## Graphical abstract

In order to take full advantage of the porous structure generated by phase separation, a bidirectional phase separation spinning method is proposed. The method enables continuous and rapid preparation of aerogel fibers without energy‐intensive additional steps, which significantly reduces energy consumption and processing time, and positions aerogel fibers as an ideal alternative for next‐generation textiles.

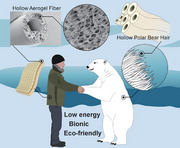

## 1 Introduction

Thermal insulation is not only about the effective use of energy and the comfort of human life,^[^ ^1^, ^2^, ^3^, ^4^ ^]^ but also about the safe operation of equipment and the sustainable development of the environment.^[^ ^1^, ^5^, ^6^, ^7^ ^]^ Aerogel is recognized as an excellent thermal insulation material due to its high porosity, resulting in extremely low thermal conductivity, and has been used in a wide range of engineering applications.^[^ ^8^, ^9^, ^10^ ^]^ Aerogels are frequently reported geometries as 3D blocks,^[^ ^10^, ^11^, ^12^ ^]^ particles (0D)^[^ ^13^ ^]^ and 2D films.^[^ ^14^, ^15^, ^16^, ^17^ ^]^ In contrast, fibers and textiles are materials that are used in virtually all of our daily activities,^[^ ^18^ ^]^ the 1D form of aerogel fibers provide excellent mechanical flexibility, structural design versatility, and dimensional adjustability.^[^ ^19^, ^20^, ^21^, ^22^ ^]^ This means that the length and radius of aerogel fibers can be easily tailored to specific requirements and further woven or stacked to create structures of any size, such as fiber mats and textiles, which can be used for thermal management at hot or cold temperature extremes. Consequently, aerogel fibers have been anticipated as promising next‐generation alternative materials for textiles and have garnered significant attention in recent years, with a wide range of applications in the fields of human body heat management, building heat management, aerospace, and polar exploration.

In order to prepare high‐performance aerogel fibers, researchers have developed various preparation strategies, such as the template method,^[^ ^23^ ^]^ the spinning method,^[^ ^19^, ^24^, ^25^ ^]^ and the emerging freeze spinning method.^[^ ^20^, ^22^ ^]^ Most of these processes require special drying methods, such as freeze drying or supercritical drying, to remove the solvent from the gel fibers to obtain the final aerogel fibers without volume shrinkage and structure cracking, which usually happens in traditional drying at ambient temperature and pressure. However, freeze drying and supercritical drying require sophisticated equipment to produce ultra‐low temperatures under vacuum and ultra‐high pressure conditions to eliminate the effects of surface tension during solvent removal,^[^ ^26^ ^]^ which results in aerogel fibers that are either challenging to achieve continuous and efficient production or often involve energy‐intensive and time‐consuming steps, thereby restricting their practical applications. Considering the significant potential application value of aerogel fibers, there is an urgent need to develop a continuous, efficient, and low‐energy consumption process for their preparation.

In this context, this work highlights a bidirectional phase separation spinning (BPSS) method, which enables fast, simple and continuous preparation of aerogel fibers at ambient temperature and pressure. The combined effect of instantaneous and delayed phase separation resulted in the formation of a compact multistage porous structure inside the fiber at room temperature, eliminating the need for additional processes like freeze‐drying or supercritical drying, which leads to a significant reduction in energy consumption and processing time. The aerogel fibers produced using this method exhibit exceptional properties, including a low density of 0.18 g cm^−^^3^, high porosity of 84%, and a wide operational temperature range spanning from −20 to 120 °C. Remarkably, when these fibers are incorporated into a thermal insulation mat, they achieve an extraordinarily low thermal conductivity of 31 ± 1.2 mW/(mK). This research introduces a novel approach to the preparation of aerogel fibers, offering energy savings and enhanced efficiency. This method is innovative, efficient, and scalable, offering a sustainable pathway for various applications, and positioning aerogel fibers as a promising contender for next‐generation textiles.

## 2 Results and Discussion

The bidirectional phase separation spinning method, as the name suggests, differs from conventional uniaxial wet spinning. The phase separation due to solvent exchange occurs at the interface between the polymer solution and water, followed by the formation of a porous structure beneath the surface layer of the polymer,^[^ ^27^, ^28^ ^]^ while the structure deeper down tends to be denser. Therefore, in order to introduce more porous structures into the fiber, the coaxial needle is used to introduce an additional polymer solution‐water interface on the inside, to interpose the sheath liquid between the core liquid and the coagulation bath to form a water‐polymer solution‐water sandwich structure from the inside to the outside (Figure S1, Supporting Information). With this, the fiber forms a hollow structure similar to polar bear hair under the effect of bidirectional solvent exchange, and the fiber can be prepared in a continuous and rapid manner. Sufficient phase separation occurs inside and outside the fiber, resulting in an extremely rich micron and submicron pore structures. Subsequently, several key governing factors of BPSS were explored, including the concentration of spinning solution (Figure S2, Supporting Information), the extrusion rate ratio of the core and sheath layers (Figure S3, Supporting Information), the ration of the coagulation bath (Figure S4, Supporting Information), and the size of the extrusion nozzle (Figure S5, Supporting Information). Eventually, the 15% wt polyacrylonitrile (PAN)/N, N ‐ dimethylformamide (DMF) was used as sheath spinning solution, and the deionized water was used as both the core spinning solution and the coagulation bath. The extrusion rate ratio of the core and sheath layers was determined to be 1:5.

**Figure 1:**
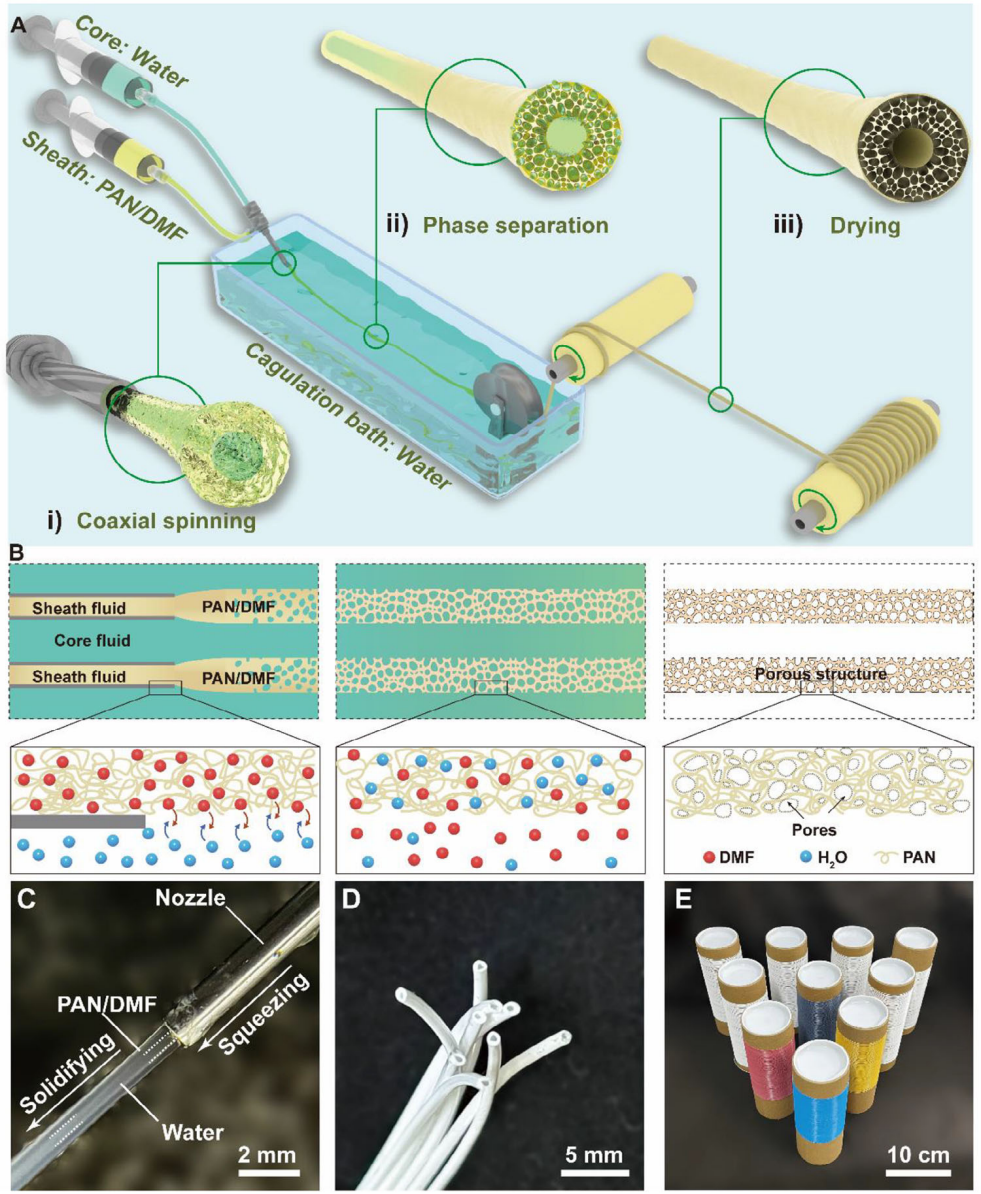
Schematic of the BPSS method and the demonstration of hollow aerogel fiber. A) The BPSS process comprises three steps: i) Coaxial spinning, ii) Phase separation, and iii) Drying. B) Demonstration of the phase separation process. C) The moment of spinning fluid extrusion. D) Photograph of the cross‐section of PAN hollow aerogel fibers (HAFs), the hollow structure is clearly visible. E) Photograph of the HAFs wrapped around the rollers.

**Figure** **1**A demonstrates the preparation process of PAN hollow aerogel fiber using the BPSS method. The BPSS method includes three procedures: coaxial spinning (Figure 1A i), phase separation (Figure 1A ii), and drying (Figure 1A iii). The BPSS method not only allows for rapid and continuous preparation of aerogel fibers, but is also more convenient and energy efficient compared to other porous fiber preparation processes. Fiber with a length of several meters can be prepared in a short time (Movie S1, Supporting Information). In principle, aerogel fibers of any length can be prepared as required.

During the spinning process, the core spinning liquid and sheath spinning solution were simultaneously squeezed into the coagulation bath continuously. Subsequently, water and the PAN/DMF solution came into contact with each other, bidirectional diffusion occurred immediately due to the large concentration difference, and led to phase separation at the same time. Pores were gradually formed inside the fiber, and contained a large amount of water and DMF, which caused the fiber to become opaque gradually. The primary fiber was dried at room temperature, water and DMF evaporated, and hollow aerogel fiber (HAF) was formed. Figure 1B fully illustrates the above process. Moreover, the spinning process did not cause changes in the crystal structure of PAN (Figure S6, Supporting Information). Figure S7 (Supporting Information) shows a photo of the coaxial nozzle and preparation process. At the moment of spinning fluid extrusion, the sheath fluid (PAN/DMF) was semi‐transparent and the internal core fluid (water) was clearly visible. Due to the extremely high mutual solubility of DMF and water, phase separation occurred rapidly and the fiber lost its translucency (Figure 1C). Figure 1D shows the photograph of the cross‐section of a cluster of fibers, the large number of internal micron and submicron pores provided innumerable gas‐solid interfaces, the diffuse reflection of the fiber was greatly enhanced, and the fiber gradually turned bright white, and the hollow structure of the fibers is clearly observed. The BPSS method in this paper is straightforward and allows for a variety of modifications using a simple process at each stage of fiber preparation, for example, multicolored fibers were prepared by co‐mixing dyes in the spinning solution (Figure S8, Supporting Information), electrically heated fibers were prepared through the modification of the fiber surface (Figure S9, Supporting Information), and hydrophobic textiles were prepared through the modification after fibers woven into a textile (Figure S10, Supporting Information). Figure 1E shows that the HAFs and HAFs and multicolored HAFs are collected on rolls neatly. The compressive and tensile properties of HAF are shown in Figure S11 (Supporting Information).

The BPSS method greatly reduces the energy consumption required for the preparation of aerogel fibers. Herein, the thermal conductivity and energy consumption of several previously reported aerogel fibers are compared. The energy consumption was assessed based on a combination of the description in the report and the paramers of commonly used equipment, specifically as shown in Table S1 (Supporting Information).^[^ ^19^, ^20^, ^22^, ^25^, ^29^, ^30^, ^31^, ^32^, ^33^, ^34^, ^35^ ^]^ The BPSS method proved to be a validated preparation strategy, and more importantly, it shows significant advantages in terms of preparation efficiency and energy consumption.

**Figure 2:**
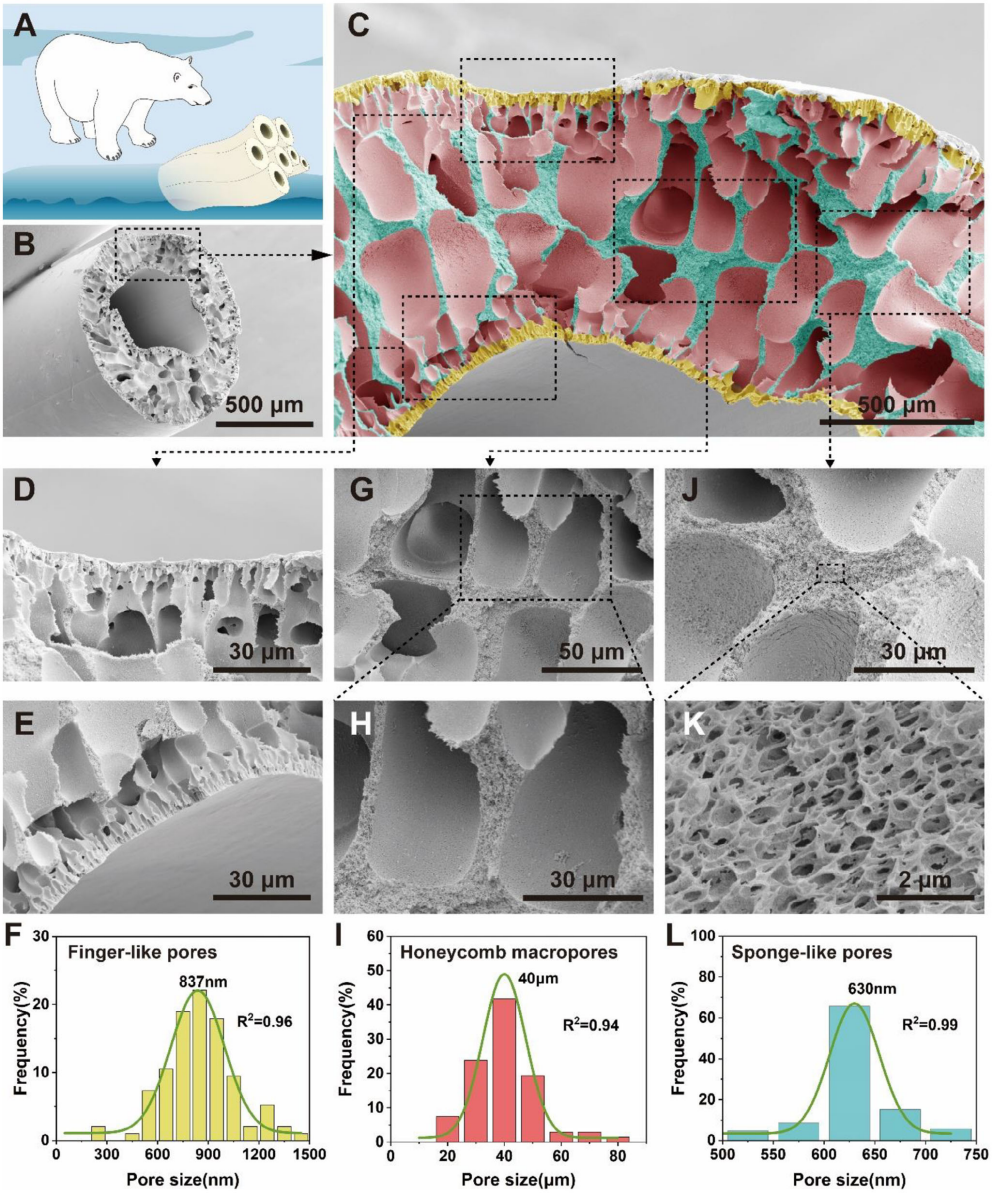
Microstructure of HAF. A) Polar bear and its hollow hairs. B) SEM image of HAF cross‐section. C) SEM image of the details of HAF cross‐section, including finger‐like pores (yellow), honeycomb macropores (red), and sponge pores (cyan). D, E), G, H), and J, K) are enlarged views of finger‐like pores, honeycomb macropores, and sponge pores, respectively. F, I, L) Size statistics of the three types of pores.

Next, the microstructure of HAF was observed to verify the effectiveness of the BPSS method on the porous structure. Deionized water is the core fluid for coaxial spinning, so the fiber has a hollow structure similar to polar bear hair (**Figure** **2**A). As shown in Figure 2B, the cross‐sectional morphology of HAF is revealed by scanning electron microscopy (SEM). The internal and external surface of the fiber is flat and smooth under the combined action of the core liquid and the solidification bath. When the PAN/DMF sheath fluid was extruded, instantaneous phase separation occurred on the interface of water and PAN/DMF due to the large solvent concentration gradient, and the fiber's internal and external surface was rapidly solidified and shaped, which resulted in a flat surface, the fiber contains a large number of pores. Three different shapes of pores are observed to be distributed inside the fiber by a further magnification of the fiber's cross‐section. As shown in Figure 2C, they are finger‐like pores (yellow), honeycomb macropores (red), and sponge pores (i. e. submicron pores, cyan). The finger‐like pores are distributed under the external (Figure 2D) and internal surface (Figure 2E) of the fiber, this is the result of transient phase separation. PAN is soluble in DMF and insoluble in water, due to the high mutual solubility of water and DMF, DMF was extracted from the sheath fluid, and the internal and external surface of the fiber was solidified rapidly. The shrinkage stress after surface curing encouraged the formation and growth of finger‐like pores, while water entered the fiber along the finger‐like pores for further solvent exchange, the average size of finger‐like pores is ≈837 nm (Figure 2F). Honeycomb macropores can be regarded as a result of the continuous expansion of finger‐like pores into the interior of the fiber. As the non‐solvent continuously entered the interior of the fiber, independent or interconnected honeycomb macropores were formed at the end of the finger‐like pores (Figure 2G). Numerous open microcells with an average diameter of 313 nm are distributed in the honeycomb macropores walls (Figure 2H; Figure S12, Supporting Information). The average size of the honeycomb macropores is 40 µm (Figure 2I). With the formation of finger‐like pores and honeycomb macropores, the concentration gradient decreased, and the diffusion rate slowed down, the form of phase separation changed from transient to delayed phase separation. Sponge pores were formed in the region between the honeycomb macropores, and the average size is 630 nm (Figure 2J,K,L).

**Figure 3:**
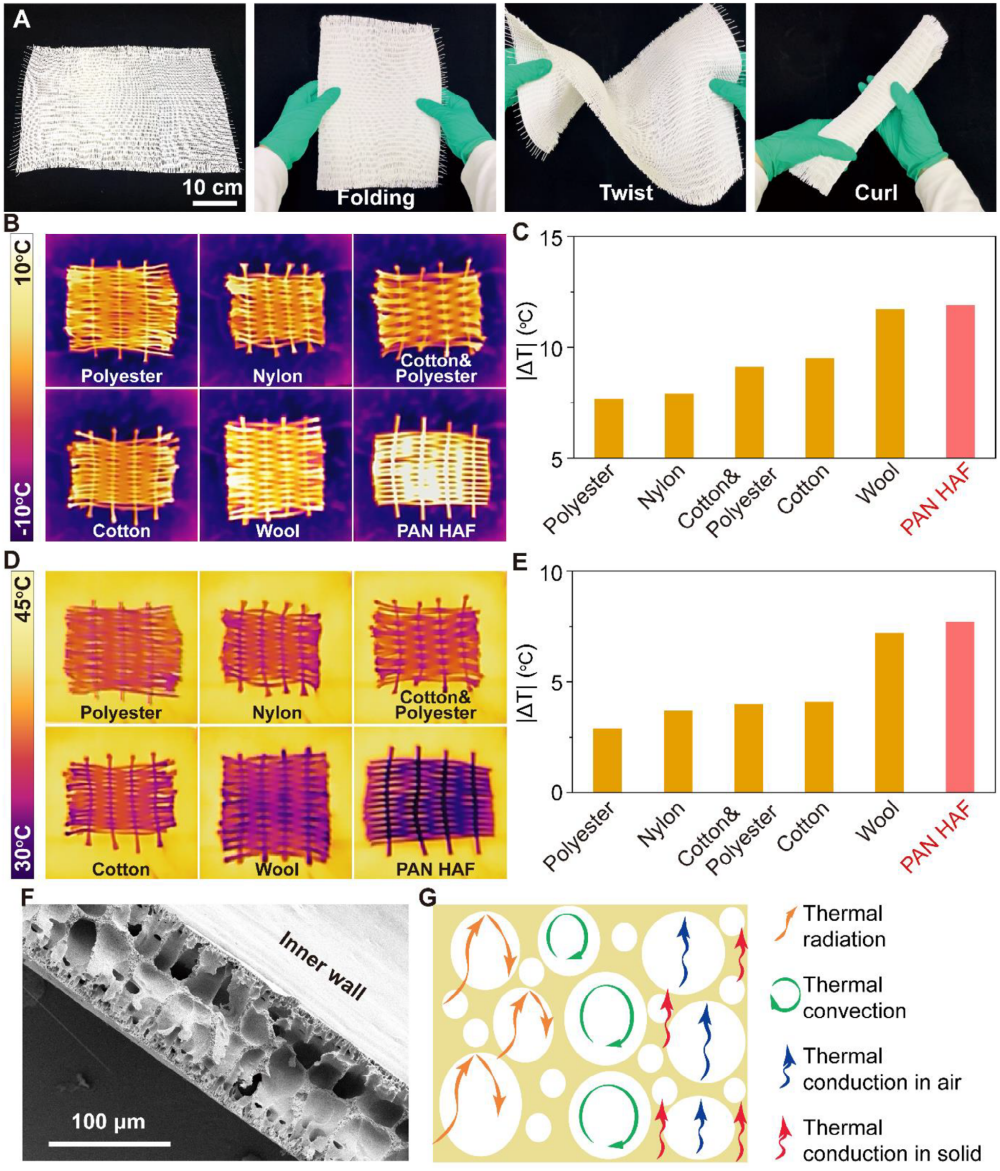
Thermal insulation properties of HAF. A) HAF textile with a length of 50 cm and a width of 40 cm can be easily folded, bent, and rolled up. B) Thermal imaging photographs of different textiles on a cooling stage at −10 °C. C) Maximum temperature difference between the textile surface and the cooling stage. D) Thermal imaging photographs of different textiles on a heating stage at 40 °C. E) Maximum temperature difference between the textile surface and the heating stage. F) Microstructure of HAF fiber in longitudinal section. G) The thermal insulation mechanism of HAF.

The microstructure of HAF proves that the BPSS method can form a porous structure. The porous structure greatly reduces the density of the fiber, which is only 0.18 g cm^−3^, and the porosity reaches 84% obtained by mercury porosimeter. As has been proven, polar bears can maintain their body temperature in the Arctic because their hollow hair structure plays a huge role. HAF, which has a similar structure to polar bear hair, has a great thermal insulating potential. Fibers were woven into textiles by plain weaving using simple weaving tools (Movie S2, Supporting Information). **Figure** **3**A demonstrates a HAF textile ≈0.5 m in length and 0.4 m in width, the textile is tight and neat. This indicates that the HAF is easy to weave and produce in large quantities, which is attributed to the high efficiency of the BPSS method. The textile can be folded, bent, and rolled up (Movie S3, Supporting Information), which suggests the fiber has good strength and flexibility, this is also supported by Figure S13 (Supporting Information). To test the thermal insulation performance, several commercial fibers with the same diameter as the HAF were selected for comparison after a simple weaving, which resulted in textiles with almost the same thickness. The above textiles were placed on the −10 °C cooling stage, and after the temperature stabilized, a series of thermal imaging pictures were taken (Figure 3B). The temperature difference between the surfaces of the textiles and the cooling stage also proved the good thermal insulating ability of HAF (Figure 3C). Similarly, the thermal images of the textiles on the heated stage at 40 °C and the temperature difference proved the same point (Figure 3D,E). In addition, the fiber exhibits high thermal stability below 200 °C as determined by Thermal Gravimetric Analyzer (Figure S14, Supporting Information).

It is difficult to test the thermal conductivity of the fiber directly, after a simple stacking, the thermal conductivity is tested using the transient plane source (TPS) method, and the thermal conductivity of the HAF plate is ≈31 ± 1.2 mW/(mK), the stacked HAF plate is also closer to the practical applications (Figure S15, Supporting Information). Theoretically, the thermal conductivity of the aerogel fiber (λ_fiber_) is determined by the sum of thermal convection (λ_conv_), solid (λ_solid_) and air (λ_air_) thermal conduction, and thermal radiation (λ_rad_). HAF has a high porosity of 84% and its multiscale porous structure is essential for reducing the thermal conductivity. The porous structure of the fiber can be visualized from the vertical section of HAF (Figure 3F). Figure 3G shows the thermal insulation mechanism of HAF, first, the thermal convection (λ_conv_) of the HAF is greatly restricted as air is blocked in the individual pore. Second, as a polymer, PAN has a special molecular structure that results in very high phonon scattering and the absence of free electrons. Therefore, the intrinsic thermal conduction of the PAN is low. In addition, the thermal conduction of air (λ_air_) is much lower than that of solid PAN (λ_solid_), the hollow structure and the numerous pores of HAF lead to significant suppression of thermal conduction^[^ ^36^, ^37^, ^38^ ^]^. Besides thermal convection and conduction, heat is transmitted mainly by thermal radiation (λ_rad_). The reflection of infrared light is significantly enhanced due to the innumerable solid‐air interfaces in the porous structure, resulting in a decrease in the penetration of thermal radiation. Among them, the effect of hollow structure and honeycomb macropores on the thermal insulation performance is mainly to hinder the solid thermal conduction. Second, the main contribution of spongy‐like pores to the thermal insulation performance is the blocking of thermal radiation. Finally, the finger‐like pores are relatively small in number, so, in general, the contribution of finger‐like pores to the thermal insulation performance is very small.

**Figure 4:**
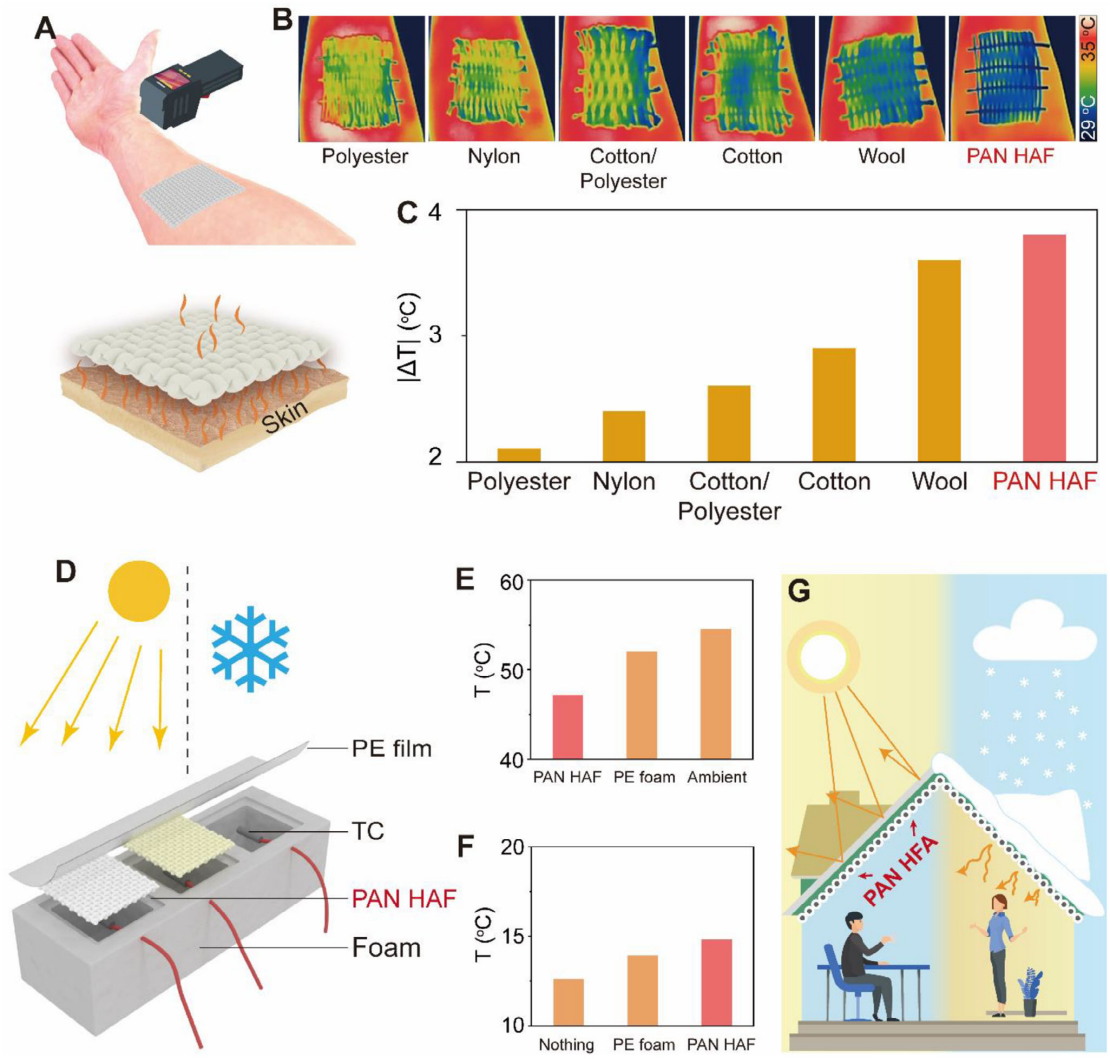
Application of HAF's thermal insulation properties. A) Schematic of the human body thermal insulation performance test. B) Thermal image of textile samples on the inner side skin of the lower arm. C) Temperature difference between skin and textile surface. D) Schematic of simulating building thermal insulation. E) and F) The temperatures of the spaces in which the individual test objects are located after stabilization. G) Conceptual illustration of HAF used as thermal insulation in buildings.

The human body can generate heat through metabolism and dissipate heat to the ambient to maintain thermal balance and normal vital signs. Textiles are the interface between the human body and the environment. Personal thermal management based on advanced textiles is becoming an effective and energy‐efficient way to achieve thermal comfort and health. As shown in **Figure** **4**A, the thermal insulation effect of the HAF textile on the human body was tested. The external surface temperature of the textiles was recorded and photographed by a thermal imager (Figure 4B). As shown in Figure 4C, the surface temperature of the HAF textile was 3 °C lower than the skin temperature, and obviously lower than other textiles. This indicates the good thermal insulation effect of HAF textile allows the heat to be stored between the textile and skin and makes it an ideal material for human thermal management.

Buildings are the main areas of human activities, and appropriate indoor temperatures are important for the maintenance of bodily functions. The popularization of heating ventilating and air conditioning system has brought about both energy consumption and harmful emissions, and there is an urgent necessity to reduce the energy consumption of buildings. The BPSS method and HAF fits this demand, on the one hand, the BPSS process is inherently low‐energy, and on the other hand, the HAF prepared by the BPSS is an excellent thermal insulation material. The use of thermal insulation materials in buildings can effectively reduce the energy consumption. Infrared radiation has a strong thermal effect, and the heat transfer from the sun to the Earth is mainly in the form of the infrared radiation. HAF can significantly isolate infrared radiation due to the presence of a large number of solid‐gas interfaces. In addition, PAN has excellent sunlight resistance and can be used as a thermal insulation layer for building thermal management. As shown in Figure 4D and Figure S16 (Supporting Information), the scene of building thermal insulation was simulated, polyethylene foam prevented the heat exchange of the surrounding environment, and PE film was covered to suppress the impact of thermal convection. Thermocouples were used to record the temperature of the chambers. Apparently, after the temperature has stabilized, the temperature inside the cavity covered with HAFs was ≈8 °C lower than ambient temperature and ≈5 °C lower than the cavity covered building thermal insulation material (Polystyrene) (Figure 4E). To simulate a winter snow scenario, the same mass of ice and water mixture was placed on the identical device, and it was obvious that the HAFs prevented the heat in the foam cavity from spreading outward (Figure 4F). These simulations show the effectiveness of the HAF as thermal insulation to resist heat gain in a hot environment and heat loss in a cold environment. By using HAF of different plies or diameters, the temperature in buildings of different latitudes, altitudes and climatic conditions can be adjusted to a comfortable range (Figure 4G). In addition, the HAF of this thermal insulation application is feasible in a wide range of ambient temperatures from −20 to 120 °C (Figures S17 and S18, Supporting Information), which makes it beneficial for the construction and related industrial sectors, and even for flexible application in daily life (Figure S19, Supporting Information).

As mentioned earlier, simplicity and continuity are important features of the BPSS method. The strategy of bidirectional phase separation is also efficient for the formation of porous structures. Herein, we performed a series of complementary validation experiment, not just for PAN, the process can be used for a wide variety of polymers. Figure S20 (Supporting Information) shows the microstructure of thermoplastic urethane (TPU) and polyvinylidene difluoride (PVDF) fiber. Likewise, they possess similar hollow and porous structures due to the bidirectional phase separation and have equally great potential for applications in other fields such as flexible sensing and radiation cooling. As shown in Figure S21 (Supporting Information), various forms of aerogels can be prepared by adjusting the shape and combination of the needles, such as aerogel films and blocks. The successful fabrication of these aerogel fibers provides new insights into the design and development of porous structures for a broad range of potential applications.

## 3 Conclusion

In this study, an ingenious bidirectional phase separation spinning method has been proposed and proved to be an attractive strategy for the preparation of aerogel fibers. This method allows continuous and efficient production of aerogel fibers without the requirement for energy‐intensive freeze drying or supercritical drying. A variety of functionalized fibers and textiles can be obtained through modification. The fibers prepared by this method have a density of 0.18 g cm^−3^, these properties being closely related to its multistage porous structure. Thermal insulation mats or textiles consisting of the fibers have a thermal conductivity as low as 31 ± 1.2 mW/(mK) and can be used flexibly to reduce heat exchange in a variety of situations, such as personal and building thermal management. The bidirectional phase separation spinning method is a potential alternative solution to develop a green and sustainable technology to meet the surge in global energy demands for heating and cooling.

## 4 Experimental Section

### Materials

Polyacrylonitrile (PAN, Mw150000) was obtained from Ispin Technology Co., Ltd., China. N, N‐Dimethylformamide (DMF) and Heptane were obtained from Shanghai Macklin Biochemical Co., Ltd., China. Hydrophobic nanoparticles (SiO_2_) were provided by Weiye Alloy Materials Co., Ltd., China. PDMS (Sylgard 184 silicone elastomer) was purchased from Dow Corning. The multi‐walled carbon nanotubes (MWCNTs, >95%, pipe diameter = 8–15 nm, tube length = 3–12 µm) were purchased from Suzhou Tanfeng Co., Ltd., China. Iron oxide pigment was purchased from Xiaoai Co., Ltd., China. All the above reagents were directly used without further purification.

### Fabrication of the HAF and Textile

The PAN hollow aerogel fiber (HAF) was fabricated through bidirectional phase separation spinning (BPSS). Fully dissolved and defoamed 15% wt PAN/DMF solution was used as sheath solution. Deionized water was used as the core spinning liquid and coagulation bath. The sheath solution and core liquid were extruded into the coagulation bath using the coaxial needle (18/25 G) at the speed of 0.50 and 0.10 mL min^−1^, respectively. A collection roller was used to collect the cured fiber. The fiber was dried at room temperature to obtain HAF, subsequently, which can be made into textile by a simple plain weave.

### Fabrication of Multicolored and Electrothermal HAF

Multicolored HAFs were prepared by mixing 4% by mass of PAN in a PAN/DMF sheath solution using the same process as before. Subsequently, the multicolored fibers were woven into multicolored textile. The HAFs were immersed in a 2% wt CNTs/DMF dispersion and held for 10 s, and then dried at room temperature to obtain electrothermal HAFs.

### Fabrication of the Hydrophobic HAF Textile

30 mL of heptane, 0.4 g of hydrophobic silica particles, 1.2 g of PDMS, and 0.24 g of silicone elastomer curing agent were mixed thoroughly and homogeneously. The HAF textile was completely immersed in the dispersion and then dried at room temperature for ≈10 min. Subsequently, it was transferred to an oven and heated to 80 °C for ≈2 h to obtain hydrophobic HAF textile.

### Characterization

For cross‐sectional observation, the HAF sample was cryo‐fractured in liquid nitrogen to get a clean cross‐section. The morphology was observed by scanning electron microscopy (Helios 5 CX Dual Beam, Thermo Scientific) at an acceleration voltage of 10 kV. All samples were sputtered with gold of 3–5 nm thickness before imaging. The diameter of the pores inside the HAF was measured on further enlarged SEM maps by ImageJ with a sample size of 100 random samples. Among them, the diameters of finger‐like pores and honeycomb macropores were measured in the short‐axis direction, and the diameters of spongy‐like pores were measured in the long‐axis direction. The histogram of the distribution after the statistics was fitted using a Gauss model. The porosity and density of HAF were measured by a mercury porosimeter (Micromeritics Auto Pore IV). All thermal images were taken by a thermal imager (Hikvision H21), and the working distance was ≈30 cm. The temperature of the sample was monitored and recorded by a thermocouple connected to the thermometer (LIHUADA DT1311). The thermo gravimetric curve was given by Thermal Gravimetric Analyzer (NETZSCH TG 209F1 Libra). Fourier transform infrared (FTIR) spectra were recorded on a FTIR spectrometer (PerkinElmer Spectrum 100). X‐ray diffraction (XRD) patterns were collected on an X‐ray diffractometer (Rigaku Smart‐Lab). HAFs were assembled into plates to test thermal conductivity using the transient planar source (TPS).

### Ethical Procedures for Vitro Experiments

The authors confirmed that there was no need to obtain Institutional Review Board approval because the volunteers only need to have brief contact with the sample at the body surface without any physical modifications nor invasive measurements on the human bodies. In addition, the volunteer was one of the authors of this study. The requirements and possible risks of these tests were fully informed before the tests. The volunteers gave written consent before participating in the experiments.

## Conflict of Interest

The authors declare no conflict of interest.

## Supporting information



Supporting Information

Movie S1

Movie S2

Movie S3

Movie S4

Movie S5

## Data Availability

The data that support the findings of this study are available from the corresponding author upon reasonable request.

## References

[1] Y. Peng , Y. Cui , Joule 2020, 4, 724.

[2] S. Wang , R. Ding , G. Liang , W. Zhang , F. Yang , Y. Tian , J. Yu , S. Zhang , B. Ding , Adv. Mater. 2023, 36, 2313444. 10.1002/adma.202313444 38114068

[3] L. Cai , A. Y. Song , P. Wu , P.‐C. Hsu , Y. Peng , J. Chen , C. Liu , P. B. Catrysse , Y. Liu , A. Yang , C. Zhou , C. Zhou , S. Fan , Y. Cui , Nat. Commun. 2017, 8, 496. 28928427 10.1038/s41467-017-00614-4 PMC5605506

[4] P. Masselot , M. Mistry , J. Vanoli , R. Schneider , T. Iungman , D. Garcia‐Leon , J.‐C. Ciscar , L. Feyen , H. Orru , A. Urban , S. Breitner , V. Huber , A. Schneider , E. Samoli , M. Stafoggia , F. de'Donato , S. Rao , B. Armstrong , M. Nieuwenhuijsen , A. M. Vicedo‐Cabrera , A. Gasparrini , S. Achilleos , J. Kyselý , E. Indermitte , J. J. K. Jaakkola , N. Ryti , M. Pascal , K. Katsouyanni , A. Analitis , P. Goodman , et al., The Lancet Planetary Health 2023, 7, 271. 10.1016/S2542-5196(23)00023-2 36934727

[5] E. Abraham , V. Cherpak , B. Senyuk , J. B. ten Hove , T. Lee , Q. Liu , I. I. Smalyukh , Nat. Energy 2023, 8, 381.

[6] J. Woods , N. James , E. Kozubal , E. Bonnema , K. Brief , L. Voeller , J. Rivest , Joule 2022, 6, 726.

[7] P.‐C. Hsu , A. Y. Song , P. B. Catrysse , C. Liu , Y. Peng , J. Xie , S. Fan , Y. Cui , Science 2016, 353, 1019. 27701110 10.1126/science.aaf5471

[8] Y. Chen , L. Zhang , Y. Yang , B. Pang , W. Xu , G. Duan , S. Jiang , K. Zhang , Adv. Mater. 2021, 33, 2005569. 33538067 10.1002/adma.202005569 PMC11468492

[9] M. Wu , H. Geng , Y. Hu , H. Ma , C. Yang , H. Chen , Y. Wen , H. Cheng , C. Li , F. Liu , L. Jiang , L. Qu , Nat. Commun. 2022, 13, 4561. 35931668 10.1038/s41467-022-32200-8 PMC9355988

[10] X. Xu , Q. Zhang , M. Hao , Y. Hu , Z. Lin , L. Peng , T. Wang , X. Ren , C. Wang , Z. Zhao , C. Wan , H. Fei , L. Wang , J. Zhu , H. Sun , W. Chen , T. Du , B. Deng , G. J. Cheng , I. Shakir , C. Dames , T. S. Fisher , X. Zhang , H. Li , Y. Huang , X. Duan , Science 2019, 363, 723. 30765563 10.1126/science.aav7304

[11] L. Li , Y. Zhou , Y. Gao , X. Feng , F. Zhang , W. Li , B. Zhu , Z. Tian , P. Fan , M. Zhong , H. Niu , S. Zhao , X. Wei , J. Zhu , H. Wu , Nat. Commun. 2023, 14, 5410. 37670012 10.1038/s41467-023-41087-y PMC10480443

[12] H. Wang , X. Zhang , N. Wang , Y. Li , X. Feng , Y. Huang , C. Zhao , Z. Liu , M. Fang , G. Ou , H. Gao , X. Li , H. Wu , Sci. Adv. 2017, 3, 1603170. 10.1126/sciadv.1603170 PMC5457032 28630915

[13] X. Zhao , W. Yao , W. Gao , H. Chen , C. Gao , Adv. Mater. 2017, 29, 1701482. 10.1002/adma.201701482 28714230

[14] Q. Zheng , L. Fang , H. Guo , K. Yang , Z. Cai , M. A. B. Meador , S. Gong , Adv. Funct. Mater. 2018, 28, 1706365.

[15] C. Fu , Z. Sheng , X. Zhang , ACS Nano 2022, 16, 9378. 35587451 10.1021/acsnano.2c02193 PMC9245345

[16] J. Lyu , Z. Liu , X. Wu , G. Li , D. Fang , X. Zhang , ACS Nano 2019, 13, 2236. 30697999 10.1021/acsnano.8b08913

[17] S. Yang , C. Xie , T. Qiu , X. Tuo , ACS Nano 2022, 16, 14334. 35994616 10.1021/acsnano.2c04572

[18] H. Wang , Y. Zhang , X. Liang , Y. Zhang , ACS Nano 2021, 15, 12497. 34398600 10.1021/acsnano.1c06230

[19] Z. Liu , J. Lyu , D. Fang , X. Zhang , ACS Nano 2019, 13, 5703. 31042355 10.1021/acsnano.9b01094

[20] Y. Cui , H. Gong , Y. Wang , D. Li , H. Bai , Adv. Mater. 2018, 30, 1706807. 10.1002/adma.201706807 29443435

[21] L. Li , G. Yang , J. Lyu , Z. Sheng , F. Ma , X. Zhang , Nat. Commun. 2023, 14, 8450. 38114508 10.1038/s41467-023-44156-4 PMC10730912

[22] M. Wu , Z. Shao , N. Zhao , R. Zhang , G. Yuan , L. Tian , Z. Zhang , W. Gao , H. Bai , Science 2023, 382, 1379. 38127754 10.1126/science.adj8013

[23] Z. Dong , C. Jiang , H. Cheng , Y. Zhao , G. Shi , L. Jiang , L. Qu , Adv. Mater. 2012, 24, 1856. 22415895 10.1002/adma.201200170

[24] G. Li , G. Hong , D. Dong , W. Song , X. Zhang , Adv. Mater. 2018, 30, 1801754. 10.1002/adma.201801754 29904953

[25] Y. Du , X. Zhang , J. Wang , Z. Liu , K. Zhang , X. Ji , Y. You , X. Zhang , ACS Nano 2020, 14, 11919. 32902257 10.1021/acsnano.0c05016

[26] X. Xu , Q. Zhang , Y. Yu , W. Chen , H. Hu , H. Li , Adv. Mater. 2016, 28, 9223. 27594204 10.1002/adma.201603079

[27] B. Wang , J. Ji , K. Li , Nat. Commun. 2016, 7, 12804. 27640994 10.1038/ncomms12804 PMC5031797

[28] M. Müller , V. Abetz , Chem. Rev. 2021, 121, 14189. 34032399 10.1021/acs.chemrev.1c00029

[29] Y. Wang , Y. Cui , Z. Shao , W. Gao , W. Fan , T. Liu , H. Bai , Chem. Eng. J. 2020, 390, 124623.

[30] M. Li , F. Gan , J. Dong , Y. Fang , X. Zhao , Q. Zhang , ACS Appl. Mater. Interfaces 2021, 13, 10416. 33595283 10.1021/acsami.0c21842

[31] J. Zhou , Y.‐L. Hsieh , Nano Energy 2020, 68, 104305.

[32] M. Li , X. Chen , X. Li , J. Dong , X. Zhao , Q. Zhang , Adv. Fiber Mater. 2022, 4, 1267.

[33] Y. Chen , C. Zhang , S. Tao , H. Chai , D. Xu , X. Li , H. Qi , Chem. Eng. J. 2023, 466, 143153.

[34] T. Xue , C. Zhu , X. Feng , Q. Wali , W. Fan , T. Liu , Adv. Fiber Mater. 2022, 4, 1118.

[35] Q. Li , Z. Yuan , C. Zhang , S. Hu , Z. Chen , Y. Wu , P. Chen , H. Qi , D. Ye , Nano Lett. 2022, 22, 3516. 35363493 10.1021/acs.nanolett.1c03943

[36] X. Zhao , Y. Liu , L. Zhao , A. Yazdkhasti , Y. Mao , A. P. Siciliano , J. Dai , S. Jing , H. Xie , Z. Li , S. He , B. C. Clifford , J. Li , G. S. S. Chen , E. Q. Q. Wang , A. Desjarlais , D. Saloni , M. Yu , J. Kosny , J. Y. Zhu , A. Gong , L. Hu , Nat. Sustain. 2023, 6, 306.

[37] F. Xiong , J. Zhou , Y. Jin , Z. Zhang , M. Qin , H. Han , Z. Shen , S. Han , X. Geng , K. Jia , R. Zou , Nat. Commun. 2024, 15, 7125. 39164288 10.1038/s41467-024-51530-3 PMC11336183

[38] F. Hu , S. Wu , Y. Sun , Adv. Mater. 2019, 31, 1801001. 10.1002/adma.201801001 30379354

