# Supplementary material for: Bidirectional Phase Separation Spinning Under Natural Drying to Prepare Aerogel Fibers for Thermal Insulation
Source: Adv Sci (Weinh). 2025 Jul 18;12(34):e05306. doi: 10.1002/advs.202505306 (PMC12442588; doi:10.1002/advs.202505306)
Supplement: Supplementary file 1 — Supporting Information [file ADVS-12-e05306-s003.docx]

Supporting Information

Bidirectional Phase Separation Spinning under Natural Drying to Prepare Aerogel Fibers for Thermal Insulation

Jiaxin Shen, Shisheng Hou, Chen Li, Kuibo Yin, Li Zhong, Hengchang Bi,* and Litao Sun*

Jiaxin Shen, Shisheng Hou, Chen Li, Kuibo Yin, Li Zhong, Litao Sun

SEU-FEI Nano-Pico Center, Key Laboratory of MEMS of Ministry of Education, Collaborative Innovation Center for Micro/Nano Fabrication, Device and System, Southeast University, Nanjing 210096, P. R. China.
E-mail: [slt@seu.edu.cn](mailto:slt@seu.edu.cn)

Hengchang Bi
Chongqing Key Laboratory of Precision Optics, Chongqing Institute of East China Normal University, Chongqing 401120, China

In Situ Devices Center, School of Integrated Circuits, East China Normal University, Dongchuan Road, Shanghai, 200241, China

E-mail: [hcbi@cee.ecnu.edu.cn](mailto:hcbi@cee.ecnu.edu.cn)


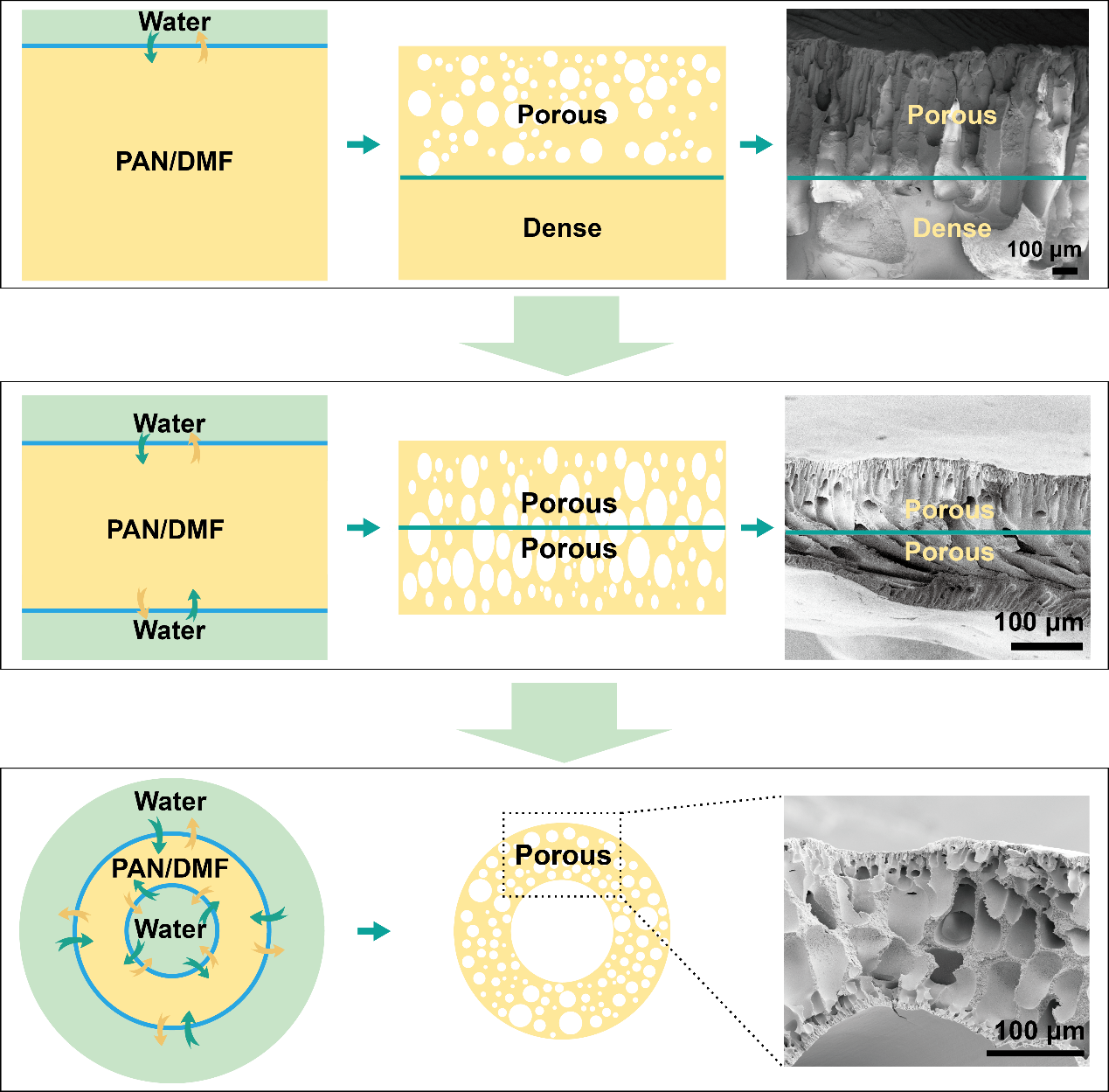


**Figure S1. Evolution of Manufacturing Strategies.**

Due to the high miscibility of water and DMF, solvent exchange occurs at the interface between the polymer solution (PAN/DMF) and water, leading to phase separation. Phase separation results in polymer solidification and the formation of a porous structure beneath the polymer surface. As the polymer surface solidifies, the rate of phase separation slows down, and the deeper structures become denser. Introducing another phase separation interface below the polymer, forming a sandwich-like water-PAN/DMF-water interface, results in bidirectional phase separation on both sides of the polymer, creating a more complex hierarchical porous structure. Coaxial spinning utilizes bidirectional phase separation to create an additional phase separation interface within the spinning flow, resulting in hollow hierarchical porous aerogel fibers.


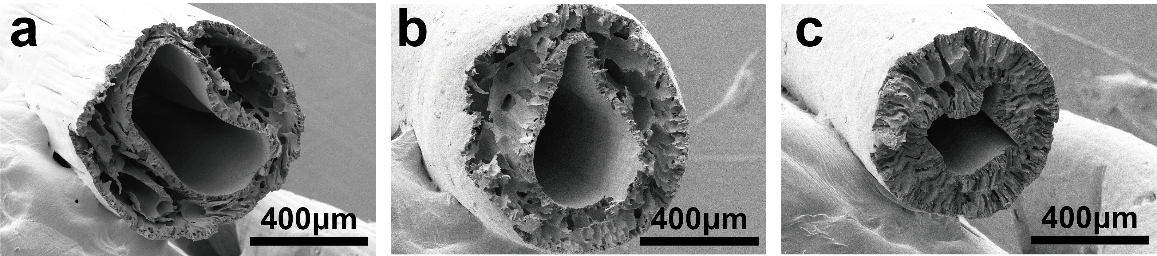


**Figure S2. SEM images of HAF's cross-sections with different PAN concentrations.**

The low concentration of spinning solution (10%) creates large cavities inside the fibers, which is a manifestation of uneven mass transfer. The strong non-solvent triggers rapid curing on the surfaces, with lagging curing on the inside, creating an inner and outer mass transfer gradient. The uncured solution shrinks to the surface due to surface tension, and eventually forms a cavity. And the high PAN concentration (20%) leads to a tendency of densification inside the fiber. In summary, the sheath solution concentration of 15% was chosen in this work.


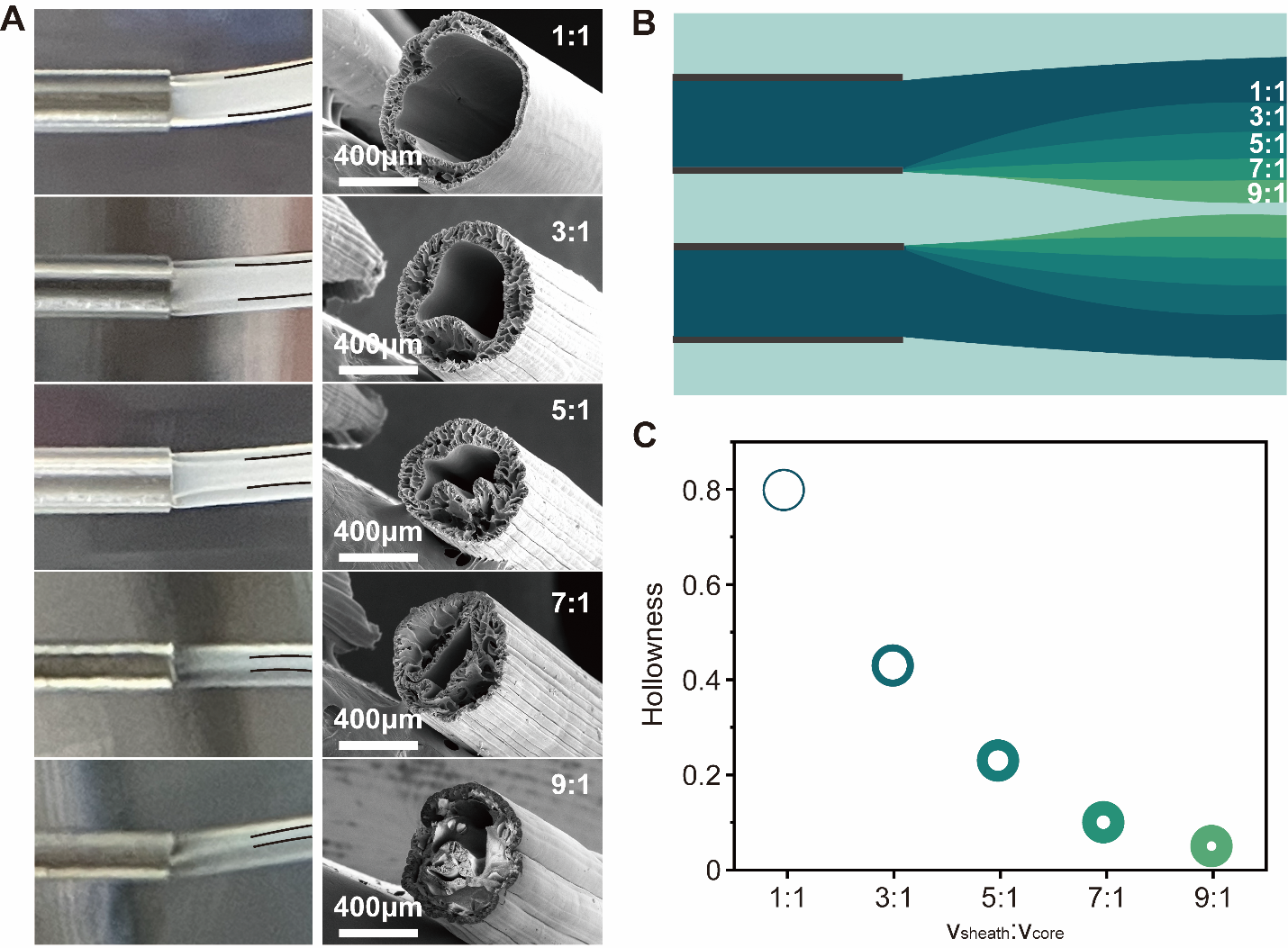


**Figure S3. Mold release expansion during extrusion.** (**A**) Photographs and SEM images of different flow ratios. (**B**) Schematic of mold release expansion. (**C**) Change of the hollowness of the fibers.

In order to rationalize the flow rate ratio of the core fluid to the sheath fluid, a series of fibers were prepared at different flow rate ratios. As shown in Figure A, there are significant differences in the cross-sectional morphology of fibers with different flow rate ratios. The hollow structure of the fiber is obvious when the ratio of core fluid to sheath fluid is 1:1, and the sheath layer is thin. However, it has been difficult to distinguish the hollow structure of the fiber when the flow rate ratio of the sheath fluid to the core fluid is 9:1. This is the result of the mold release expansion effect due to the shear flow, as shown in Figure B, with the increase in sheath fluid flow rate, the space for the core fluid is further compressed. The ratio of the hollow area (S_h_) to the fiber cross-sectional area (S_f_) was defined as hollowness: H = S_h_/S_f_. Figure C illustrates the quantitative relationship between hollowness and flow rate ratio. In this study, we consistently determined the velocity ratio of sheath flow to core flow to be 5:1.

**
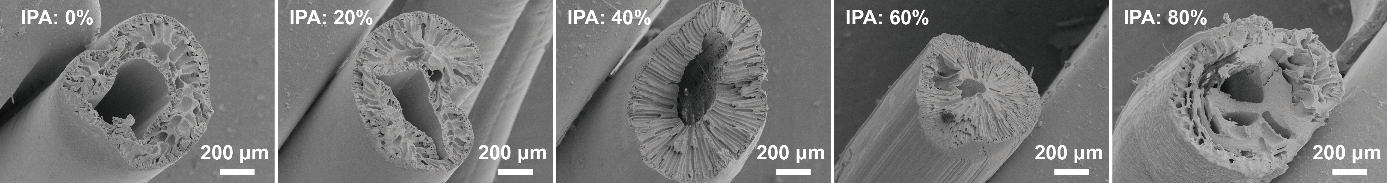
**

**Figure S4. SEM images of cross sections of HAFs prepared in coagulation baths with different IPA concentrations.**

Non-solvent diffusion, especially of water, plays an important role in the solidification of PAN solutions and the formation of pore structures. Mixing different types of non-solvents and solvents as coagulants can change the Hansen solubility parameter, which can effectively regulate the pore structure formed. The inward diffusion of IPA is much slower than that of water, and the higher the content of IPA in the coagulation bath, the slower the phase separation of the PAN solution. As can be seen in the figure, with the increase of IPA concentration, the pore structure inside the fiber, especially the honeycomb macropores gradually regionally reduced, and the structure of the fiber is gradually dense.

**
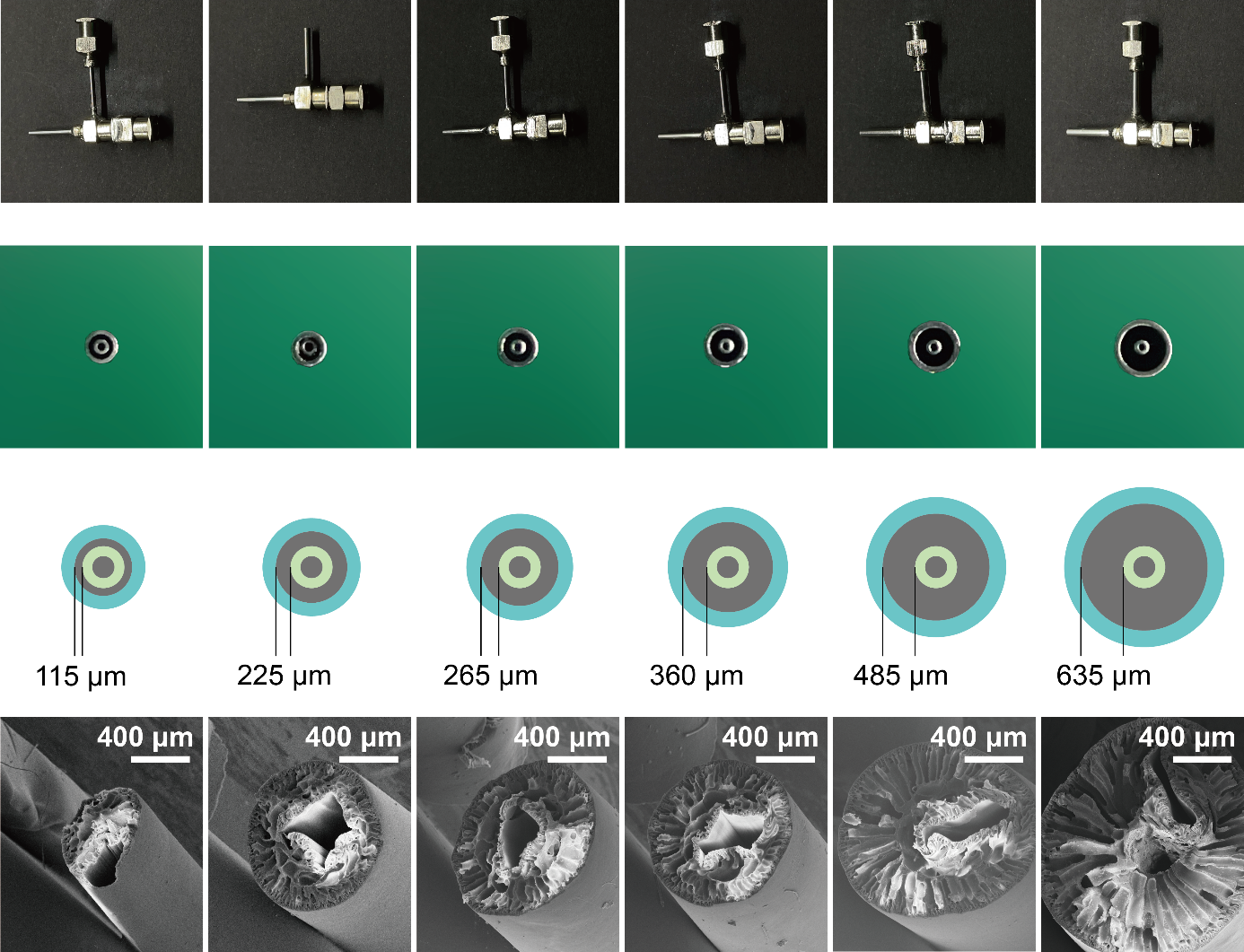
**

**Figure S5. HAFs prepared with different sizes of coaxial needles**


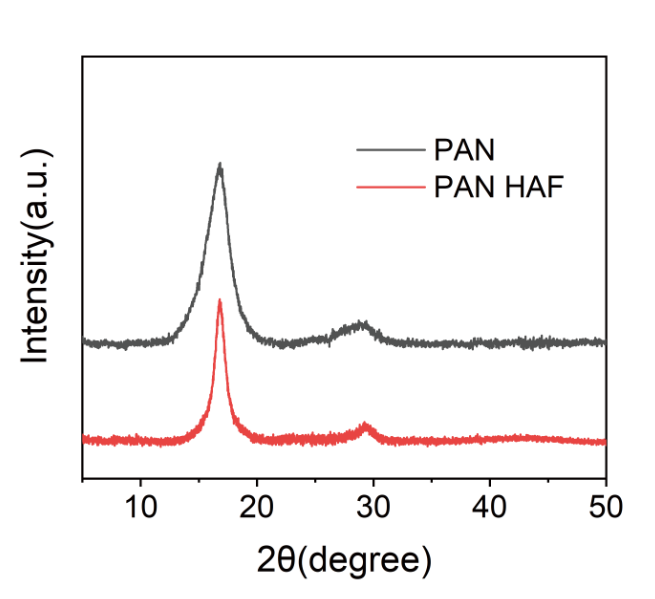


**Figure S6. The XRD patterns of PAN and PAN HAF.**

As shown in the Figure S6, the X-Ray Diffraction (XRD) patterns of PAN and PAN HAF have no change in peak position and intensity, and the consistency of the peak positions implies that the type of crystal structure inside the material has not been fundamentally changed during the preparation from PAN to PAN HAF. In addition, the half-slit width of PAN HAF is slightly narrowed, which may be caused by the unavoidable mechanical stress on the fiber during the spinning process. In the spinning process, the fiber material undergoes a complex mechanical processing and is subjected to a variety of stresses, such as tensile and shear. These mechanical stresses can have a significant effect on the PAN molecular chain. The application of stress induces the PAN molecular chains, which may be disordered, to gradually orient themselves in the direction of the stress. The orderly arrangement of molecular chains leads to more regular intermolecular interactions and provides more favorable conditions for the crystallization process. The crystallinity of the material is increased. The increase in crystallinity means that the proportion of the crystalline part of the material increases, and the crystal structure becomes more regular and orderly. This change in microstructure is directly reflected in the half peak width of the XRD spectra.


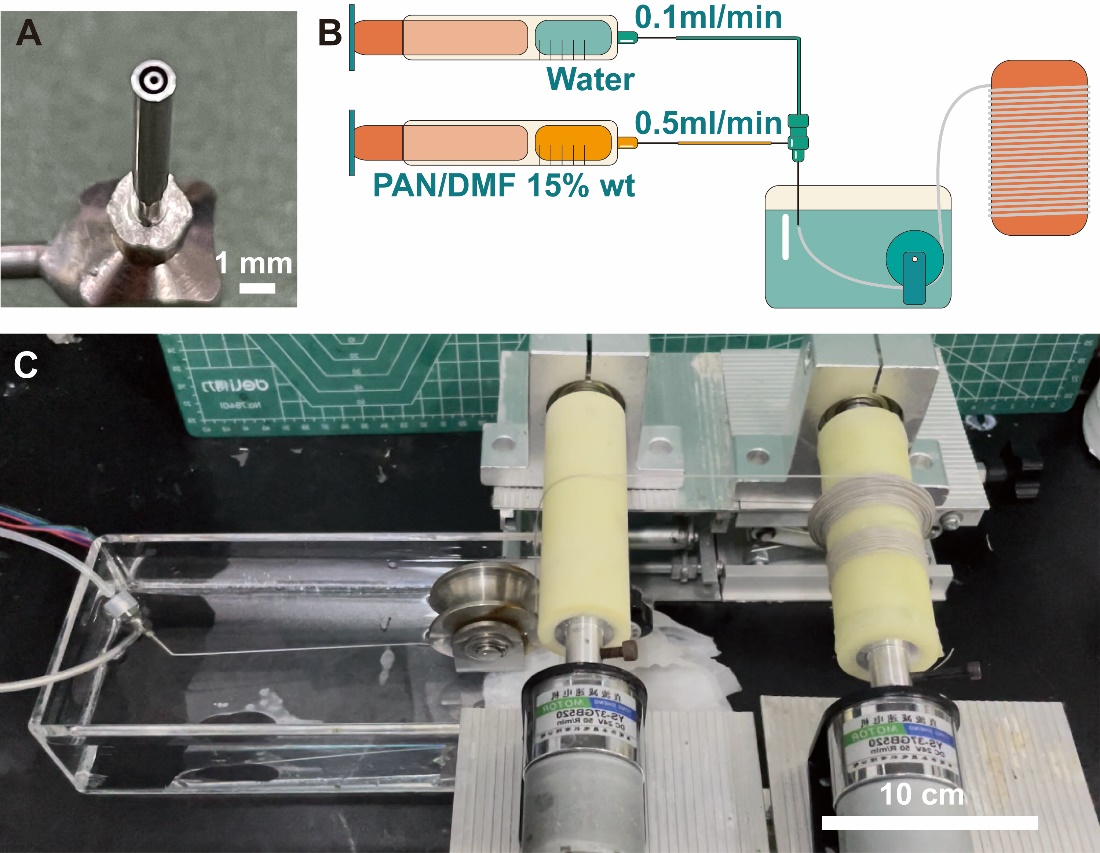


**Figure S7. The parameters of spinning.** (**A**) Coaxial nozzle. (**B**) Flow rates of core and sheath fluids. (**C**) Photo of the spinning process.

The nozzle in this work consists of a coaxial internal 25-gauge (G) needle and an external 18-gauge (G) needle respectively (Figure A). Figure B shows the detailed spinning parameters of the fibers, and Figure C shows a physical view of the spinning.


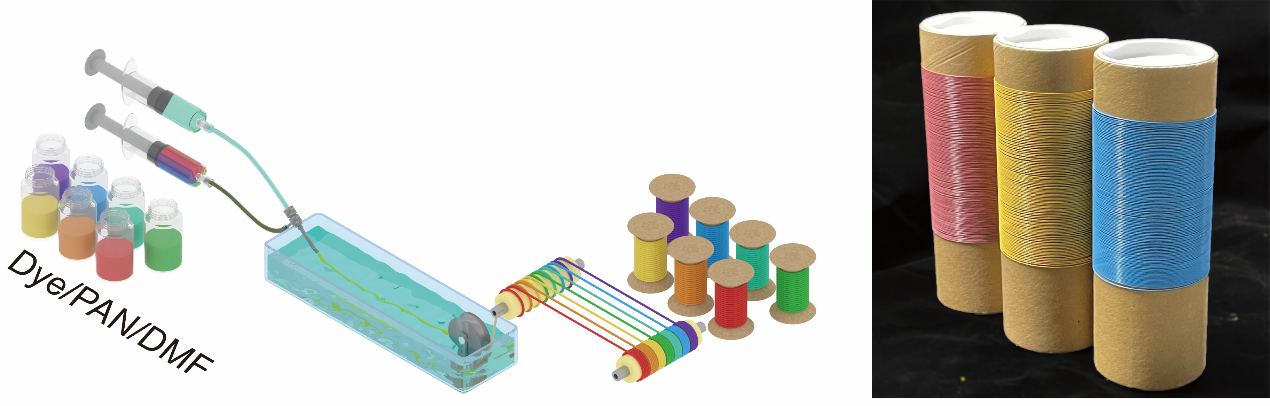


**Figure S8. Preparation and demonstration of multicolored fibers.**

Multicolored PAN HAFs can be easily prepared by mixing iron oxide pigments into the spinning solution using the same process parameters as before, which makes PAN HAF to be used in various fields, such as civilian multicolor clothing and camouflage clothing for military uniforms.


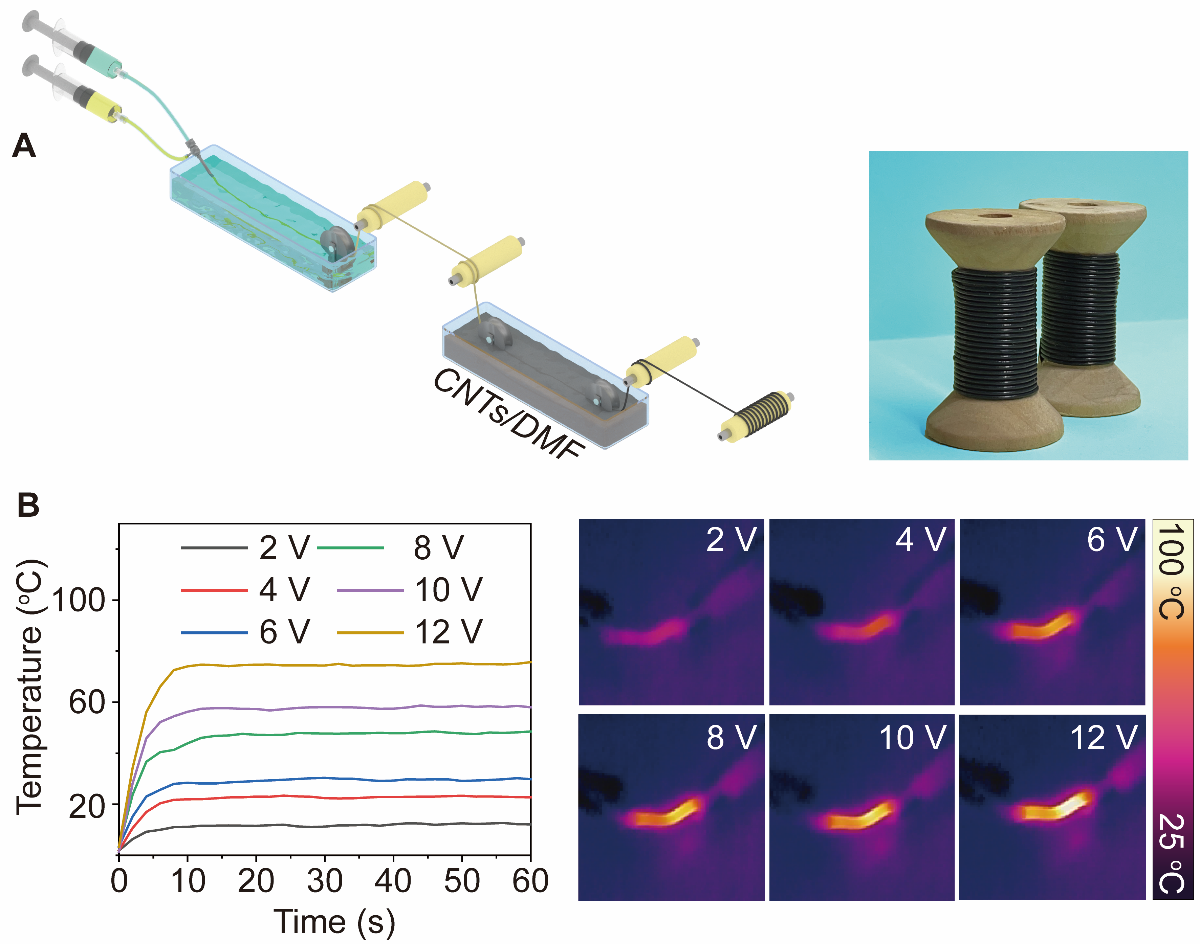


**Figure S9. Electrothermal fibers.** (**A**) Preparation and demonstration of electrothermal fibers. (**B**) The performance of electrothermal fibers.

Herein CNTs were used to improve the electrical conductivity of PAN HAF to realize electric heating. The swelling behavior of PAN in DMF was utilized to lock CNTs onto the PAN HAF surface and built up a continuous conductive network (Figure A). The fiber was connected to an electrical circuit. The temperature of the fiber increased rapidly and stabilized at 2, 4, 6, 8, 10 and 12 voltages (Figure B). Notably, the voltage for electric heating is far below the safety threshold for the human body, it can help the body to maintain temperature safely in cold environment.


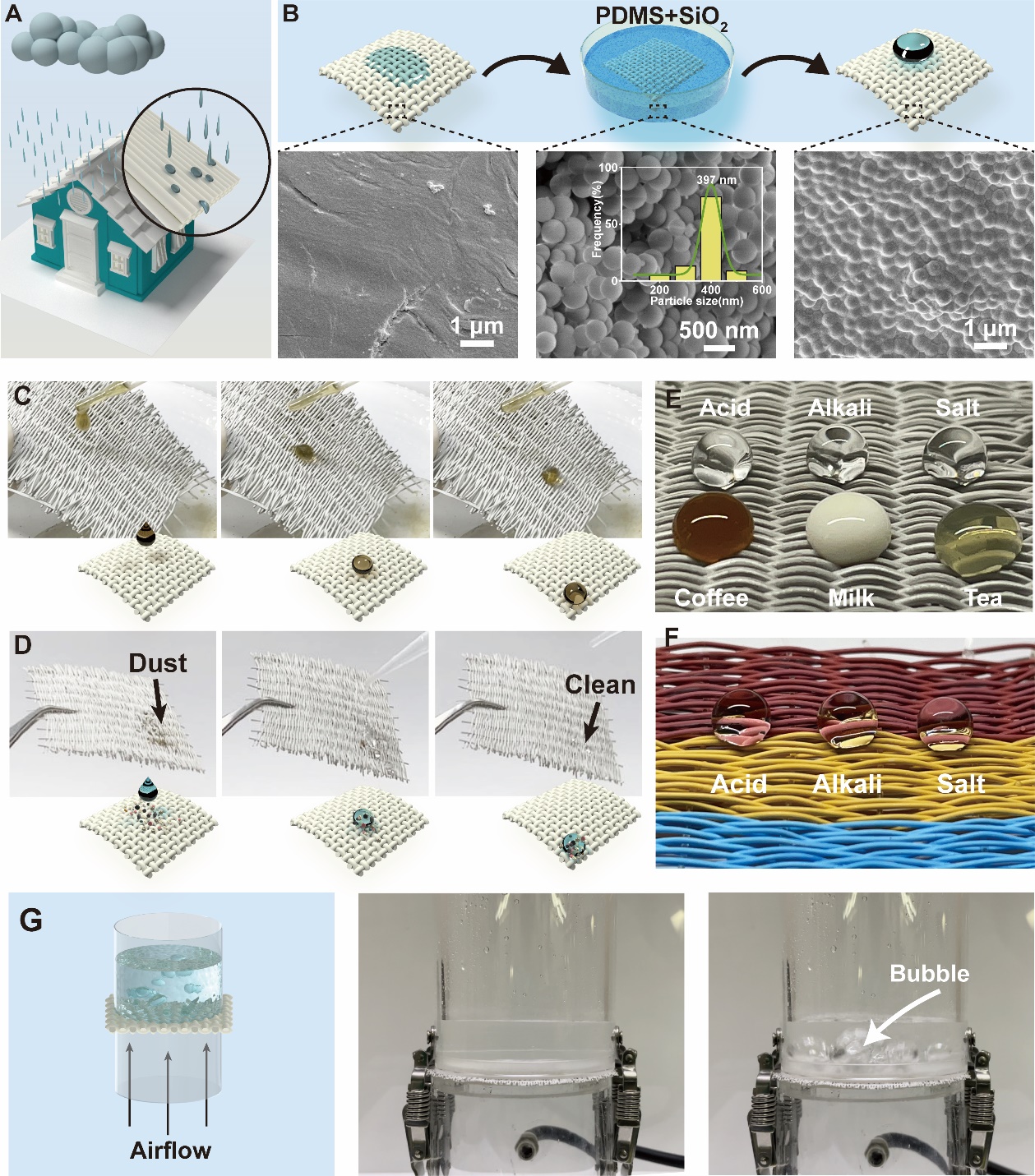


**Figure S10. Hydrophobicity and self-cleaning of HAF.** (**A**) Concept demonstration of the hydrophobicity of HAF for building thermal insulation. (**B**) Preparation process. Photograph and schematic of (**C**) the hydrophobicity test and (**D**) the self-cleaning test. (**E**) Spread of water with different pH and ingredients over HAF textile and (**F**) trichromatic HAF textile. (**G**) Waterproof and breathable of HAF textile.

Hydrophobicity is particularly important to the durability and practicality. Due to the hydrophobicity, the liquid on the surface rolls off quickly as droplets because of the surface tension. On the one hand, good hydrophobicity can ensure that water droplets do not penetrate into the interior of the textile to affect the thermal insulation effect, on the other hand, rolling droplets can absorb dust, so that the fiber mat has the ability to self-cleaning, the durability of the fiber is enhanced, reducing the output of human and financial resources (Figure A). The key to hydrophobicity is low surface energy and rough surface, The hydrophobic modification process of HAF is shown in Figure B. PDMS was used as a low surface energy material and adhesive, and the rough surface was provided by the woven texture and hydrophobic silica particles attached onto the surface of the fiber, the average size of silica particles is about 397 nm. The surface of the fiber changed from flat to uneven and granular. As shown in Figure C, sewage did not stain the textile, and it kept the droplet shape sliding down. Figure D demonstrates the self-cleaning process of the textile. Dust was carried away by liquid droplets to enable the self-cleaning of the textile (Movie S4). Notably, Figure E demonstrates that its hydrophobicity would not deteriorate either with pH (1, 7 and 13, adjusted by hydrochloric acid, sodium sulfate and sodium hydrate, respectively) or the ingredient (coffee, milk or tea). Additionally, multicolored fibers can also be hydrophobically modified in the same way (Figure F). Demonstration of the waterproofness and breathability of HAF textile using the device shown in Figure G. The water above did not fall due to the hydrophobicity of the textile, and bubbles were observed in the water above when the air was blown in below, which proves the breathability of the textile (Movie S5).

**
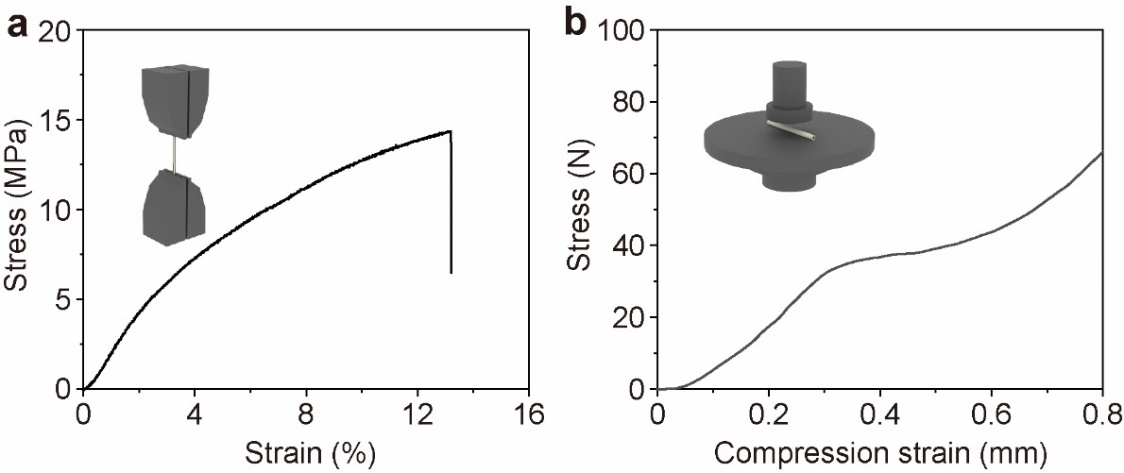
**

**Figure S11. Mechanical properties in tensile and compression testing of HAF**

The molecular chain of polyacrylonitrile contains polar cyano group (-CN), and there are strong hydrogen bonds and dipole-dipole interactions between molecules, so the molecular chain of PAN has low activity. The internal pore structure acts as a “stress buffer unit”, absorbing energy through the plastic deformation of the pore edges during stretching, resulting in an elongation at break of more than 13%. In addition, the pore structure can improve the overall stiffness and stability, in compression can be through the plastic deformation of stress sharing, in the radial compression of the fiber, the fiber can withstand a lot of stress. At a compressive deformation of about 80% of the fiber diameter itself, a compression stress of up to 60 N can be achieved.


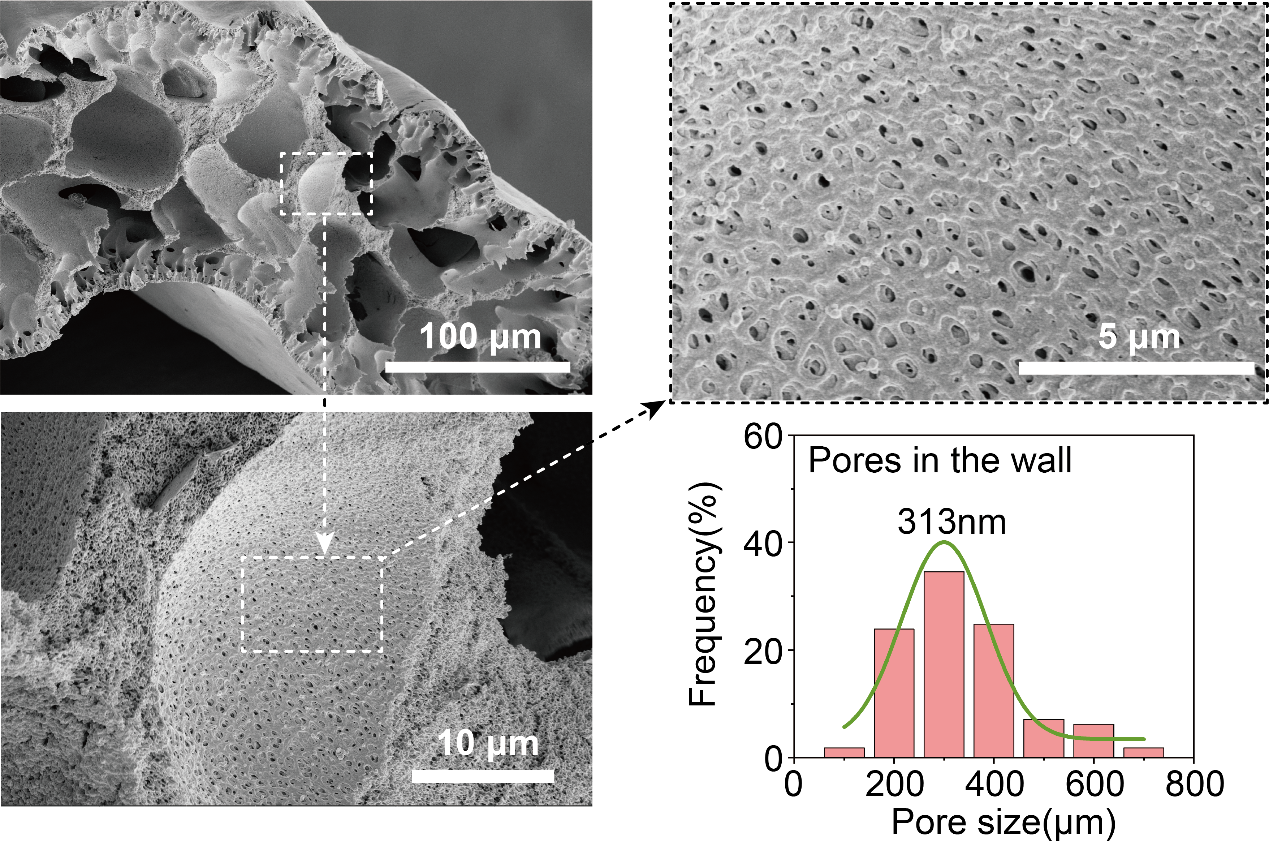


**Figure S12. Pores in the wall.**

The numerous open microcells in the pore walls with an average diameter of 313 nm. These may be channels for water to enter the sponge-like pore area after honeycomb macropores are formed.


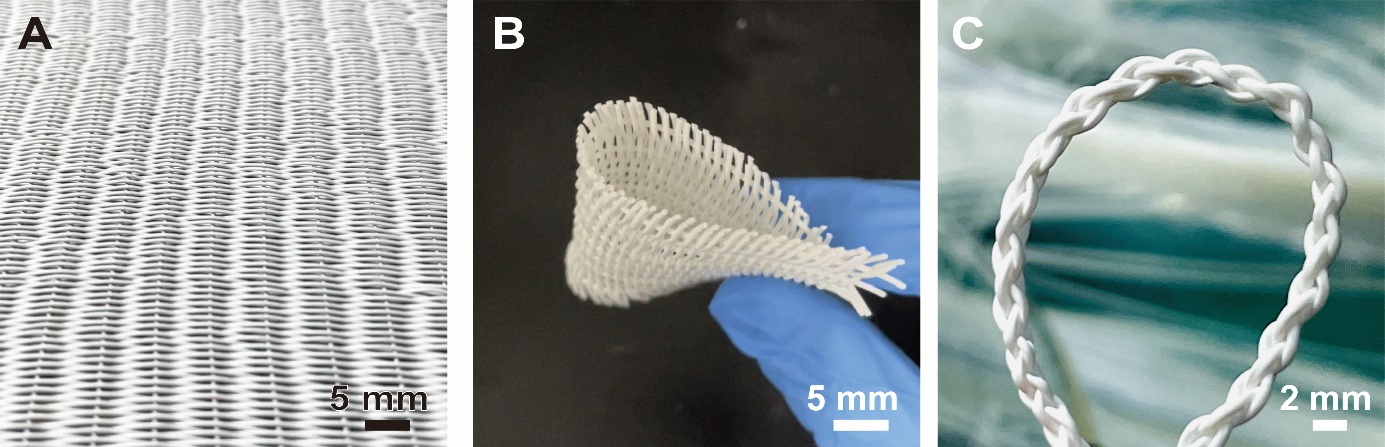


**Figure S13. Detail and flexibility of HAF textiles.** (**A**) Photograph of the details of the textile, the textile is neat and dense. The bending of small pieces of textile (**B**) and braid of PAN HAF (**C**) indicate that the PAN HAF has good flexibility.


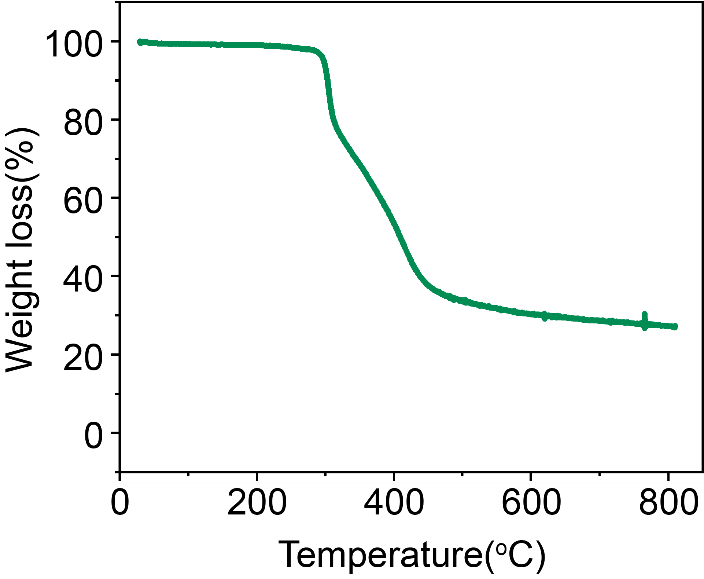


**Figure S14. TGA curve for the PAN HAF.**

The fibers have good thermal stability and can meet the heat resistance needs of the human body and building thermal insulation.


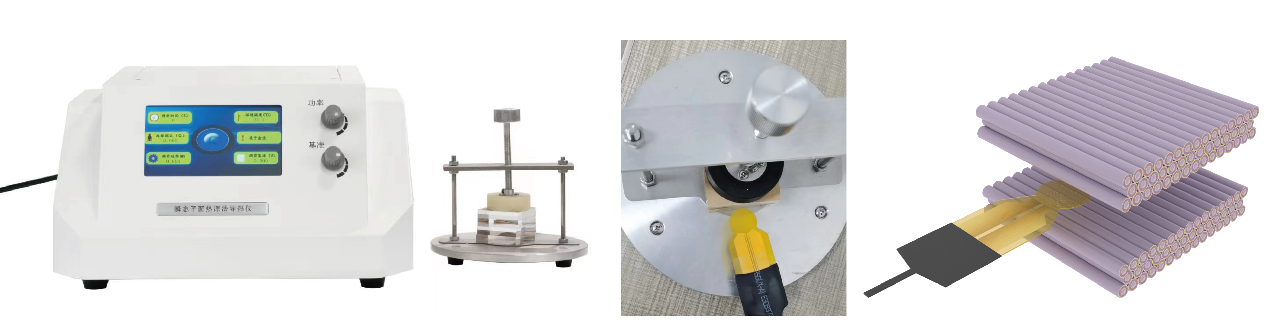


**Figure S15. Thermal conductivity testing of HAF.**

The thermal conductivity of fibers is tested using the Transient Plane Source (TPS) method. The fibers are stacked closely together because it is difficult to test the single fiber. The stacked form is closer to its application scenarios. The probe is placed between two groups of fibers as both a heat source and a temperature sensor, the thermal conductivity is obtained by recording the temperature versus time.


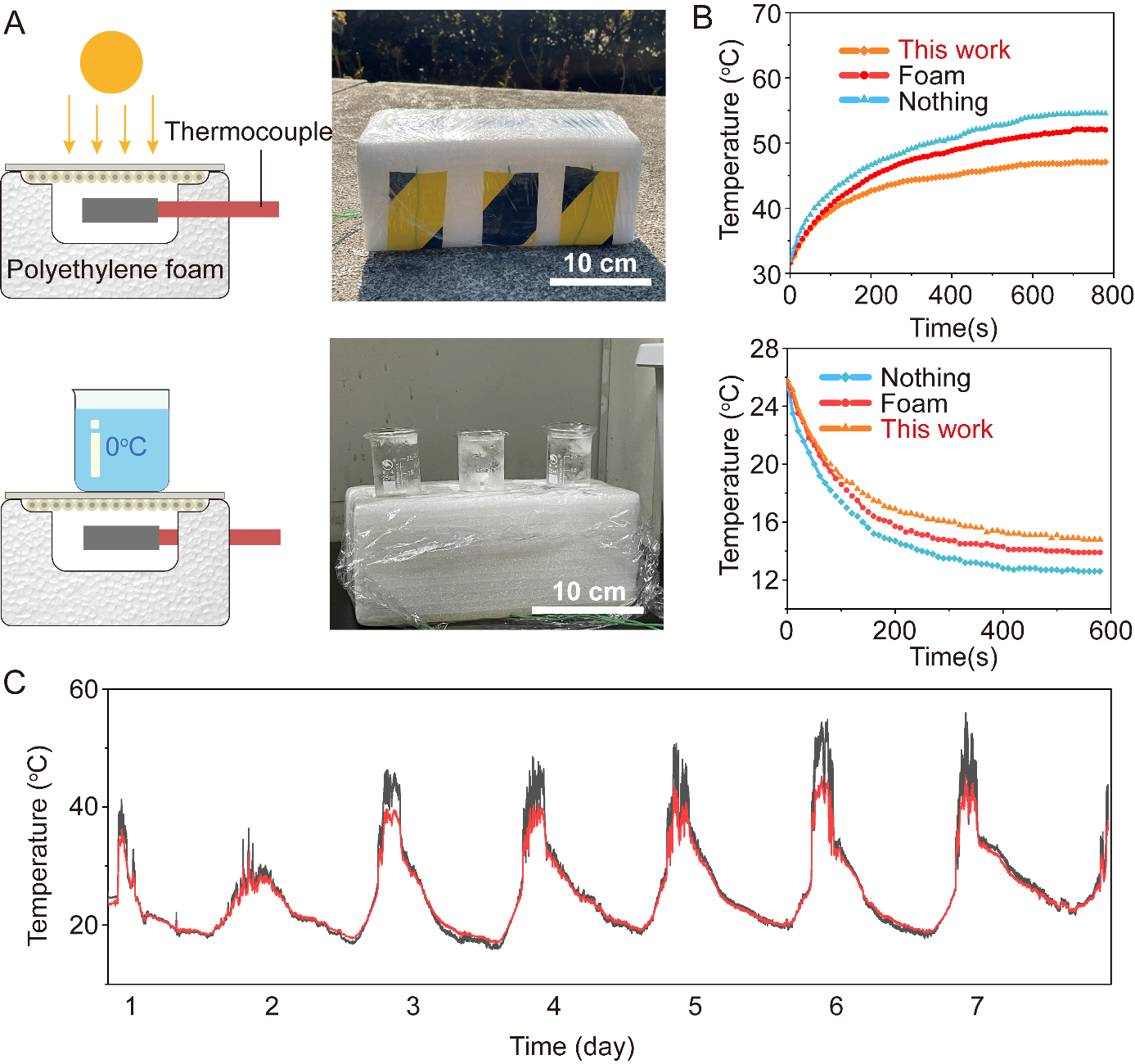


**Figure S16. Applications of HAF in the construction sector.**

Figure A show the schematics and photographs of the simulated building insulation. Cavities were cut out of polyethylene foam to simulate the interior space of the building. The thermocouples were placed in each cavity and did not come into contact with anything. Two of the cavities were covered with PANHAFs and polystyrene building thermal insulation foam of the same thickness respectively. As a comparison, the last cavity was not covered with any object to measure the air temperature. PE film was covered over all cavities to inhibit the effect of heat convection. The temperature changes of the experimental setup under sunlight and under a mixture of snow and ice are shown in Figure B. It is clear that the HAF prevents the exchange of heat between the inside and outside of the foam cavity. In order to better verify the long time stability of HAF as a building insulation material, we tested its temperature for about seven days, and the results are shown in Figure C. In the daytime, especially when sunshine was sufficient, HAFs played the most advantageous role, and after the sunset, the temperature dropped sharply, and the cavity covered with HAFs could still be insulated for a period of time, but since there was no heat source inside the cavity, it eventually reached thermal equilibrium with the outside. This shows that in building insulation, the main way of HAF insulation is the obstruction of thermal radiation.


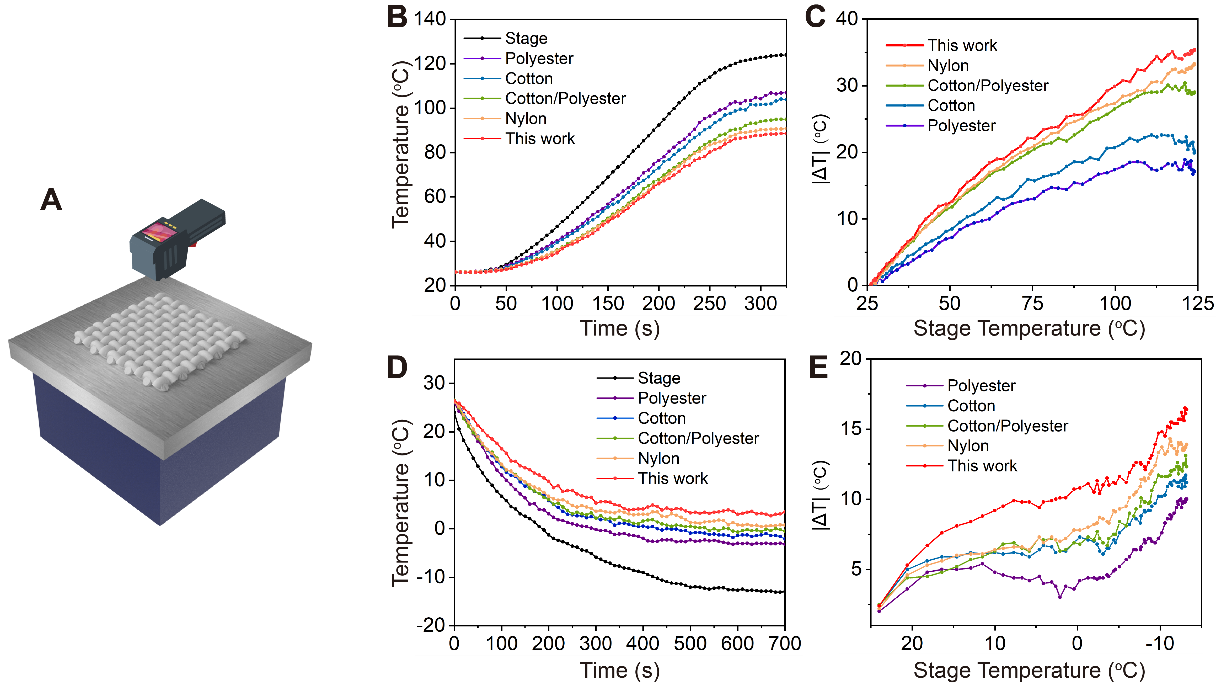


**Figure S17. Thermal insulation of HAF textile compared with commercial textiles.**

The thermal insulation of HAF textile was observed during a continuous temperature change process. Different textiles were placed on the same heating or cooling stage (Figure A). Figure B and C show the heating process, and Figure D and E show the cooling process. The outer surface of the HAF textile always maintains the maximum temperature difference from the heated or cooled stage.


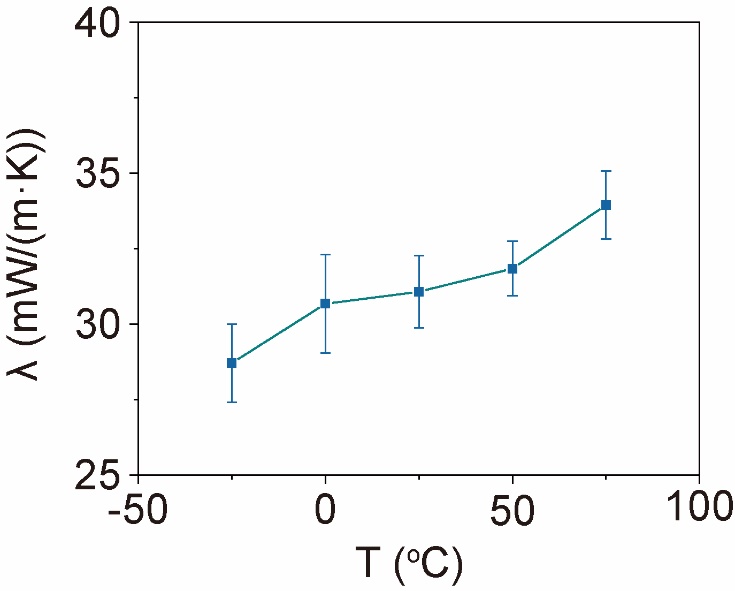


**Figure S18.** Thermal conductivity of HAF at different temperatures

The thermal conductivity of HAF fiber mats was tested in the interval from -25 to 75 ℃. At low temperatures (i.e., below the glass transition temperature, the molecular vibration is the main contributor to thermal conductivity, and the thermal conductivity of HAF shows an upward trend as the temperature increases to enhance the vibration.


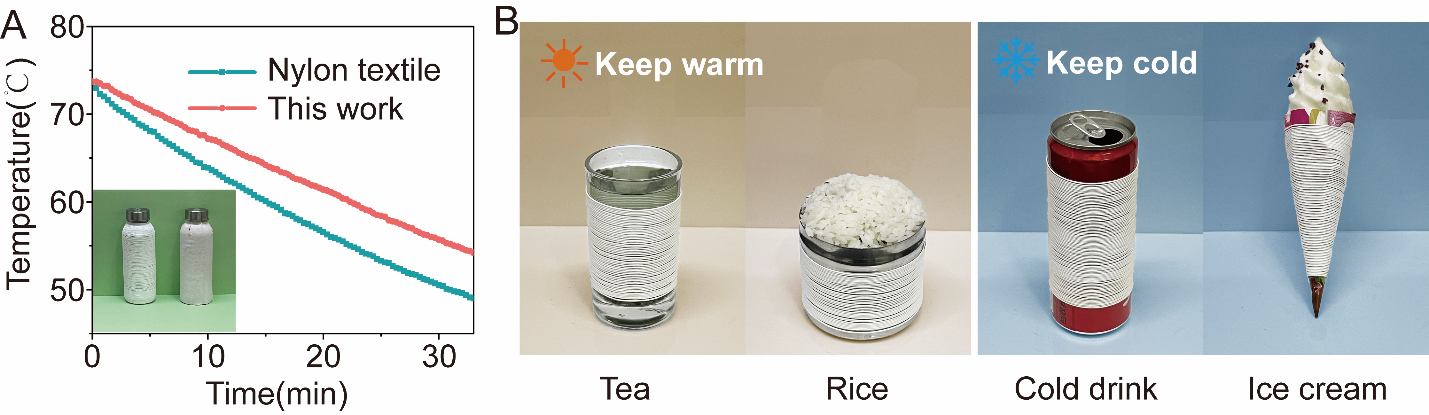


**Figure S19. Applications of HAF in the in daily life.**

Due to its flexibility and adaptability, the PAN HAF can be easily assembled for various daily scenarios, such as beverage sleeves. As shown in Figure A, two identical glass bottles were filled with hot water of the same temperature and volume, the same thickness of PAN HAFs and nylon fabric were tightly wrapped around the outside of the bottles, and thermocouples were placed in the same position in the bottles to measure the temperature change of the water, the temperature change curves show the thermal insulation performance of PAN HAFs. PAN HAFs can be applied to surfaces of various curvatures and materials such as glass, metal and paper to retain heat and keep cold (Figure B). These simulations show that PAN HAF has the potential to be applied flexibly in various scenarios where thermal insulation is required to resist the intrusion or dissipation of heat.


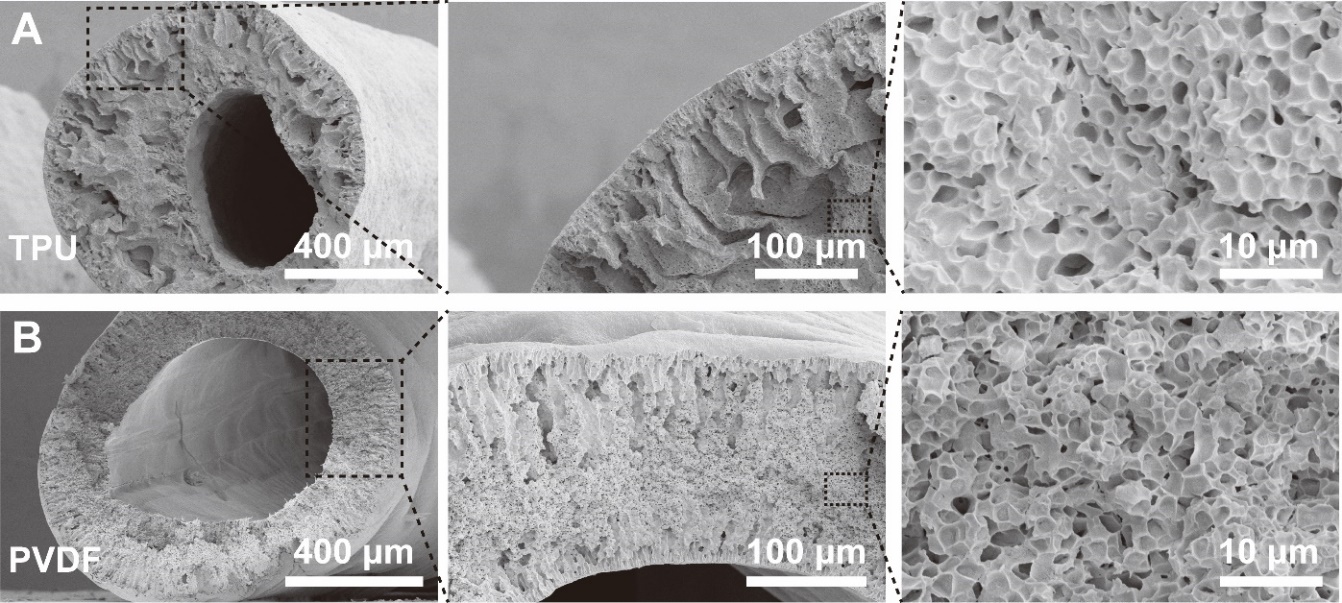


**Figure S20. SEM images of the cross-section of TPU hollow aerogel fiber and PVDF hollow aerogel fiber.**

The bi-directional phase separation spinning method is highly expandable. A variety of polymer fibers were prepared utilizing this method, such as TPU aerogel fibers and PVDF aerogel fibers.

TPU aerogel fiber was prepared by the following method:

The TPU was purchased from BASF, model 1185A. It was dissolved in DMF and configured into a solution with a concentration of 25% as the sheath solution, which was spun using a coaxial needle of 18 G-25 G. The core solution and coagulation bath were deionized water. The extrusion rate of the sheath solution was 0.5 ml/min. and the core solution was 0.06 ml/min.

PVDF aerogel fiber was prepared by the following method:

The PVDF was purchased from Ispin Co. Ltd., and the molecular weight was 400,000. It was dissolved in DMF and configured into a solution with a concentration of 15% as the sheath solution, which was spun with a coaxial needle of 18G-25G. The core solution and coagulation bath were deionized water. The extrusion rate of the sheath solution was 0.5 ml/min. and the core solution was 0.1 ml/min.

Each aerogel fiber has its own attractive properties, such as TPU aerogel fiber can be used for flexible sensing due to its good elasticity (Figure A), while PVDF can be used for radiative cooling due to the strong radiation generated by the bending vibration of the C-H and C-F groups in the atmospheric transparent window coupled with its porous structure (Figure B).


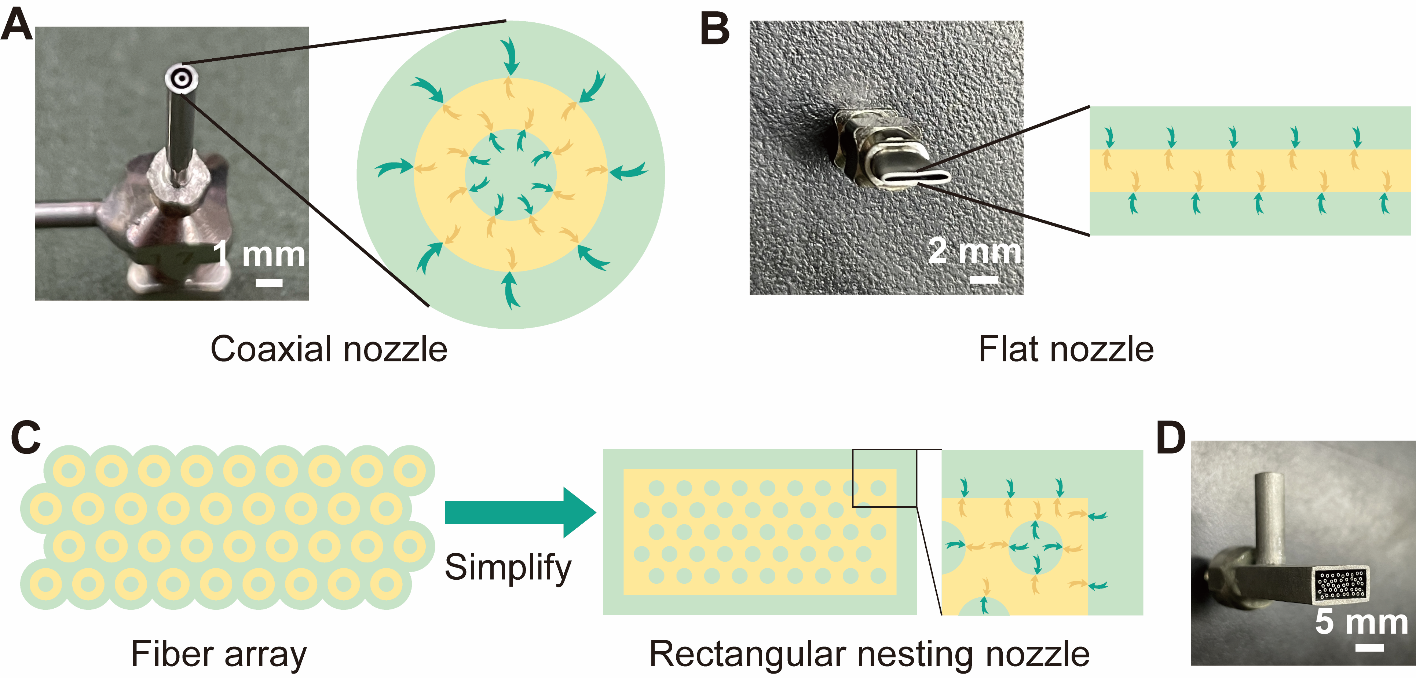


**Figure S21. Expansion of bi-directional phase separation.**

The strategy of introducing phase separation interfaces within the polymer is not limited to fibers (Figure A). A flat extrusion nozzle can be used to produce aerogel sheets (Figure B), while introducing dense phase separation interfaces within a rectangular extrusion nozzle can directly produce aerogel blocks (Figure C and D).

**Table S1 Previously reported process and energy consumption**

| Reference in main paper | Process | Time (h) | P (W) | Fibre Diameter | Energy consumption |
| --- | --- | --- | --- | --- | --- |
| 29 | Freeze-drying | 24 | 1000 | 1442 | 141030.78 |
| 22 | Freeze-drying | 48 | 1000 | 960 | 125013.20 |
| 30 | Freeze-drying | 18 | 1000 | 725 | 26737.49 |
| 31 | Freeze-drying | 192 | 1000 | 200 | 21703.68 |
| 32 | Freeze-drying | 18 | 1000 | 620 | 19553.66 |
| 33 | Supercritical drying | 9 | 2000 | 580 | 17112.00 |
| 34 | Freeze-drying | 8 | 1000 | 700 | 11077.92 |
| 19 | Freeze-drying | 24 | 1000 | 350 | 8308.44 |
| 25 | Supercritical drying | 9 | 2000 | 370 | 6963.83 |
| 20 | Freeze-drying | 48 | 1000 | 220 | 6565.36 |
| 35 | Freeze-drying | 8 | 1000 | 50 | 56.52 |
| **This work** | **Natural Drying** | **No requirement** | **0** | **648** | **0** |

Here, the energy consumptions of the preparation methods in previous studies on aerogel fibers are compared, all of them involving lyophilization or supercritical drying. A rough calculation of the energy consumption per meter of aerogel fiber preparation was made using the following rules. The power of a commonly used lyophilizer is about 1000 W. The maximum amount of material that can be processed at one time is about 1 kg of water content, and the drying time is estimated to be 8 hours according to the limit of lyophilizer performance. Meanwhile, taking the supercritical dryer commonly used in the laboratory as an example, its power is about 2000 W, and the average processing time for a material with a moisture content of 1 kg is about 9 h. In addition, some preparation methods involve processes such as mixing, extruding, freezing, transferring, etc., and the energy consumptions related to these processes are ignored because the energy consumption is much lower than that of lyophilized drying and supercritical drying. The bidirectional phase separation spinning process in this study requires only natural drying. The energy consumption per meter of aerogel fiber is demonstrated in the last column of Table S1. High cost can certainly result in high thermal insulation performance, but the negative economic effect is also huge, so it seems that the BPSS method provided in this paper is a cost-effective and effective method.
